# Supplementary figures and images for: Rigid versus flexible: The effect of destination revitalization policy on tourists’ travel behavioral intention
Source: PLoS One. 2026 May 7;21(5):e0348289. doi: 10.1371/journal.pone.0348289 (PMC13152149; doi:10.1371/journal.pone.0348289)

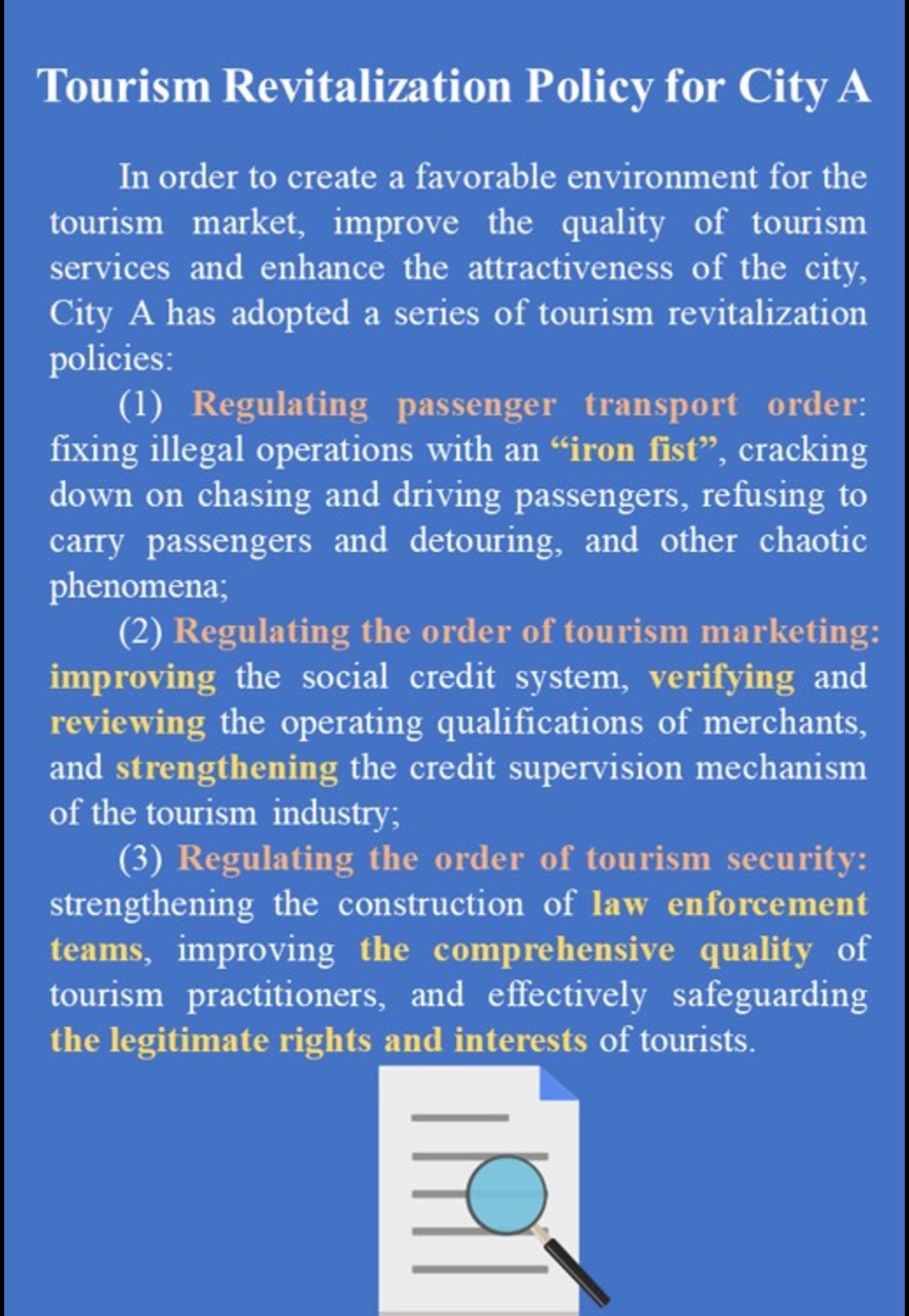

Supplement: S1 Fig — (TIF) [file pone.0348289.s001.tif]

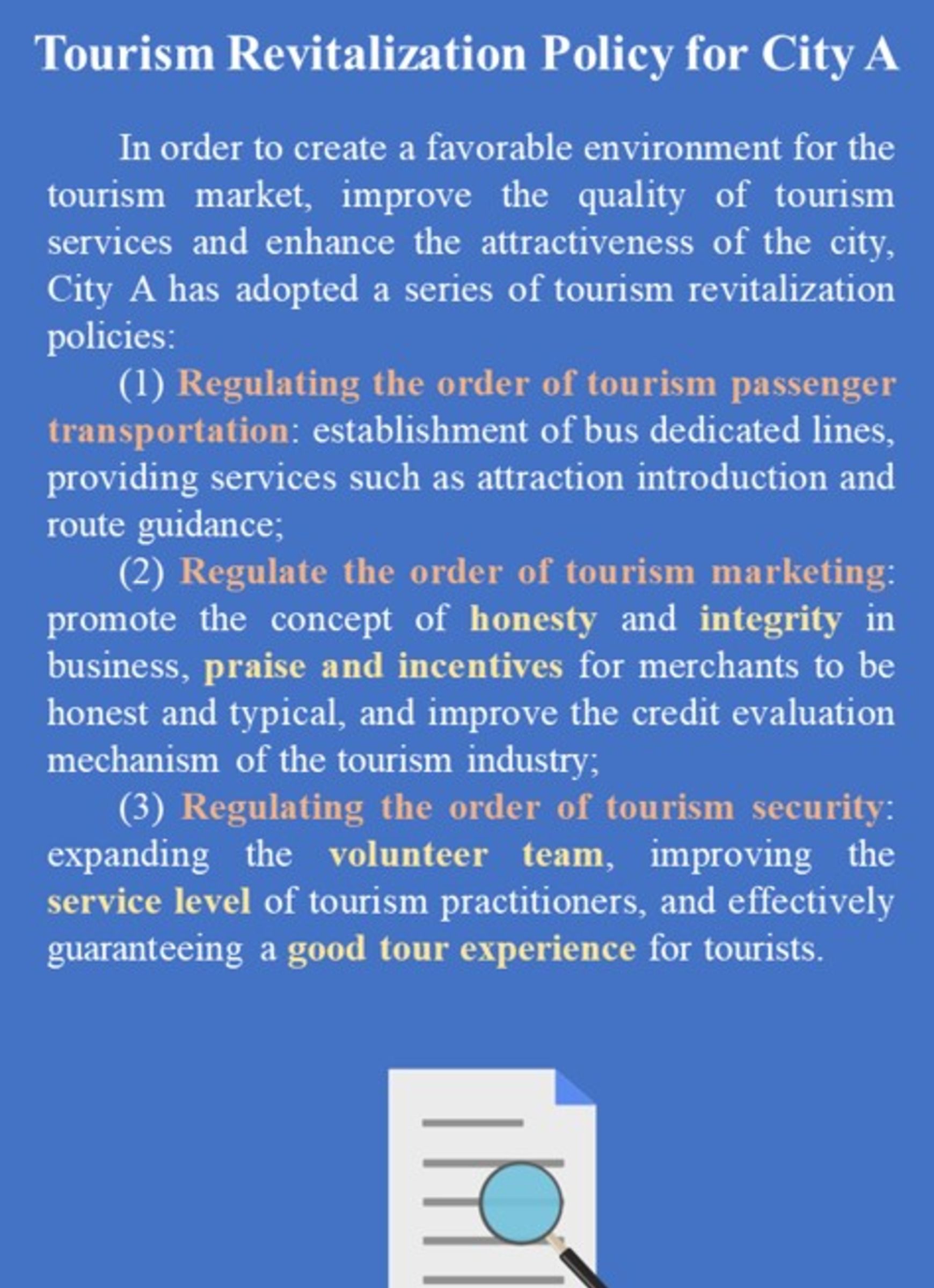

Supplement: S2 Fig — (TIF) [file pone.0348289.s002.tif]

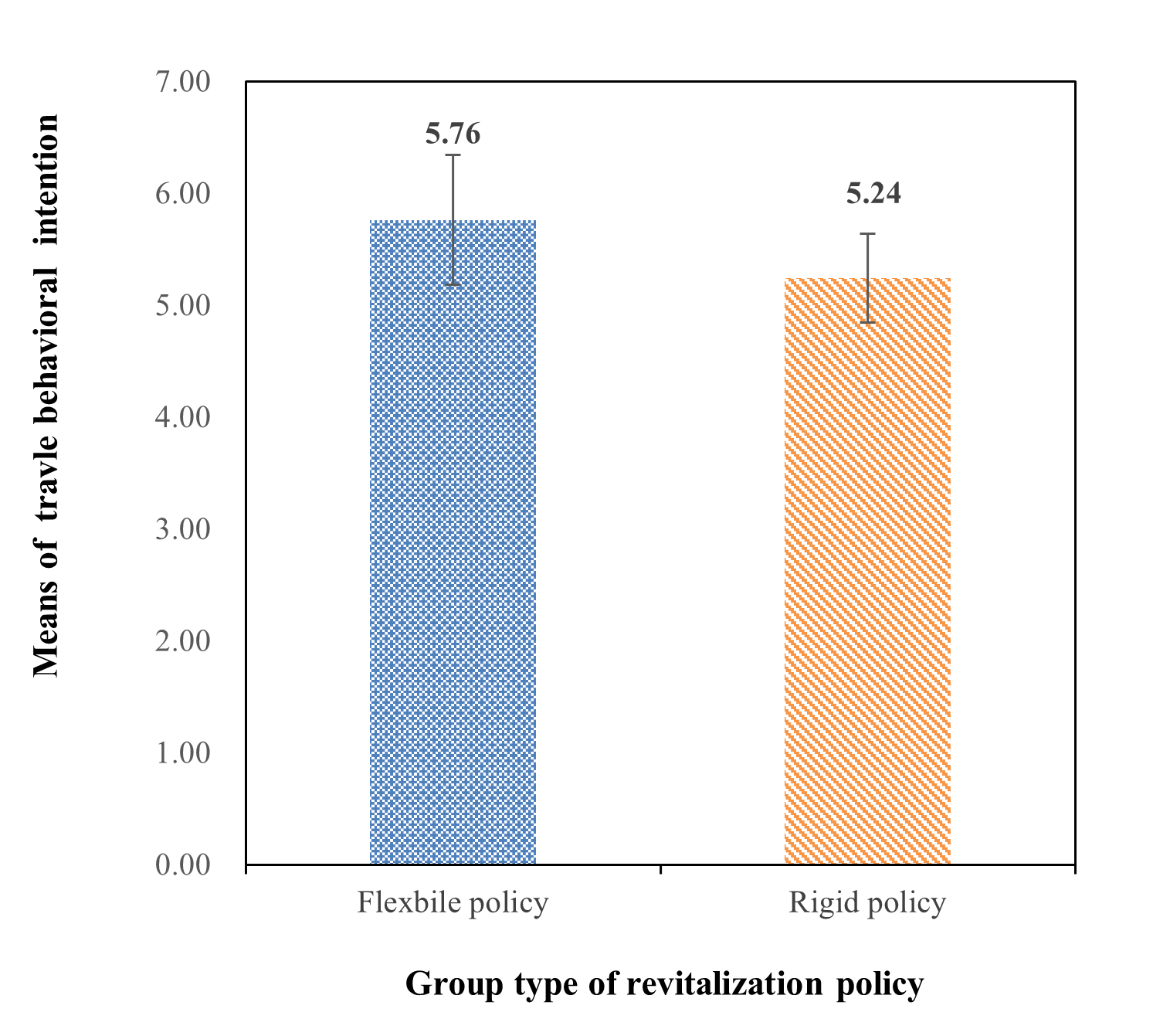

Supplement: S3 Fig — (TIF) [file pone.0348289.s003.tif]

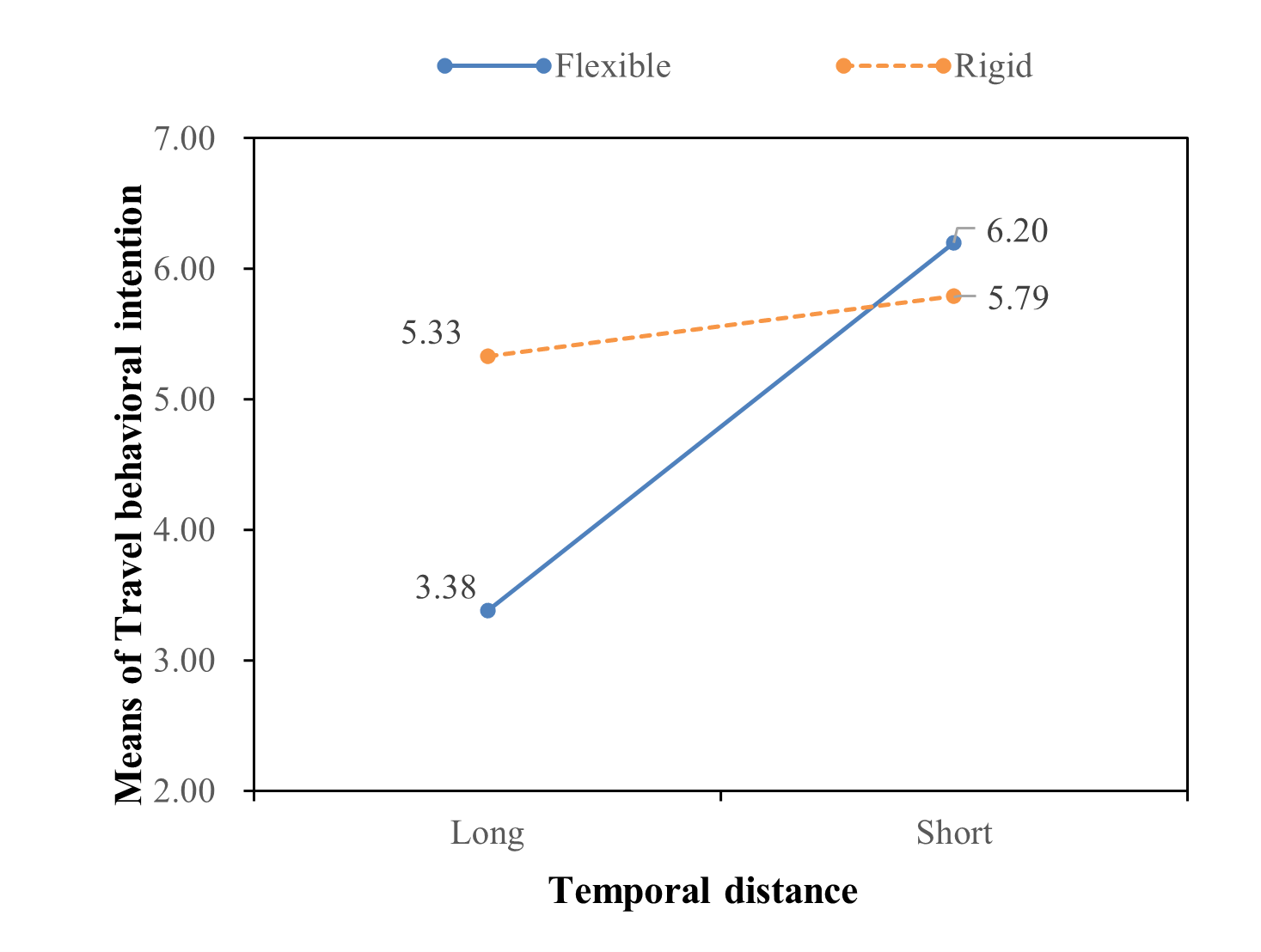

Supplement: S4 Fig — (TIF) [file pone.0348289.s004.tif]

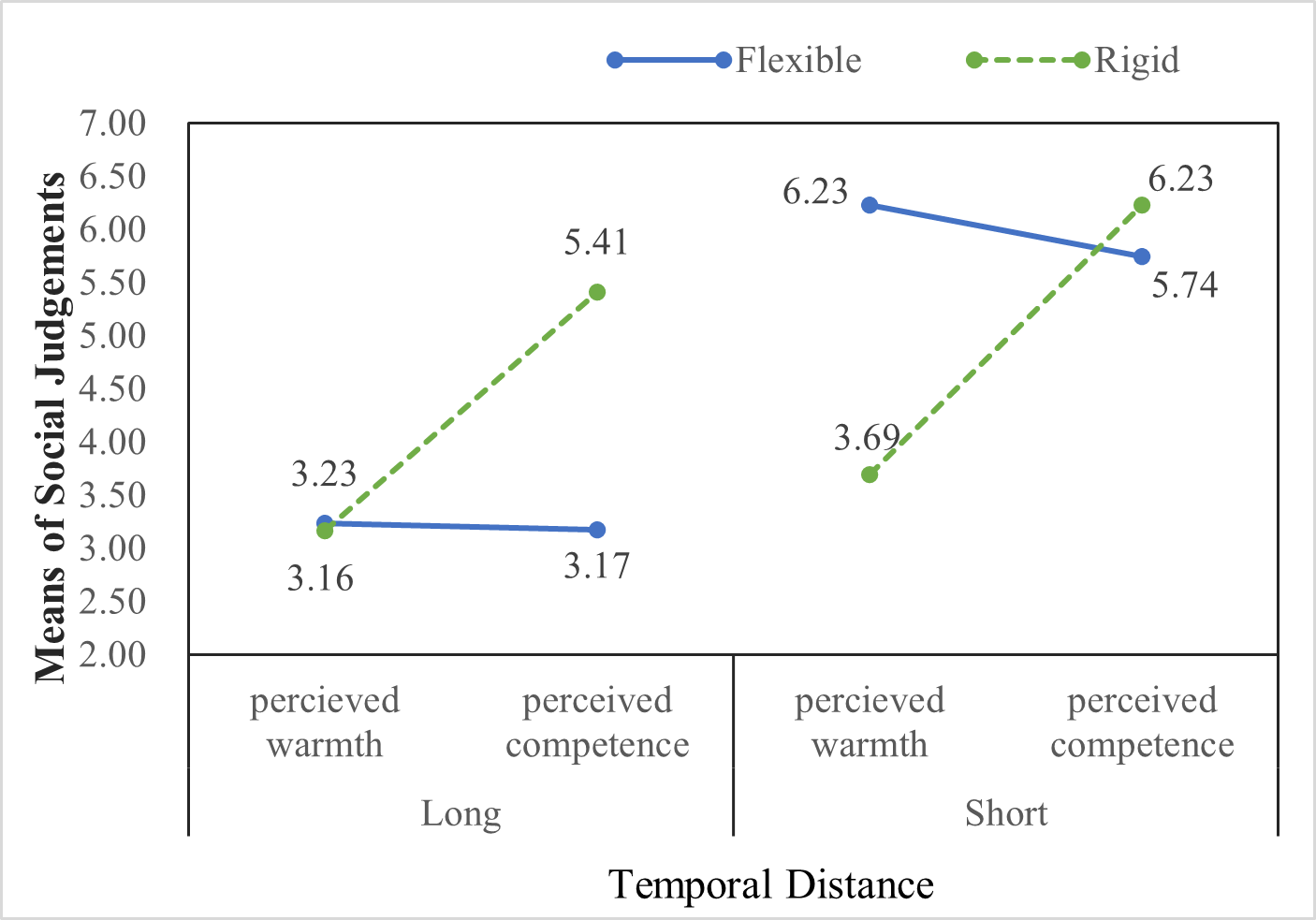

Supplement: S5 Fig — (TIF) [file pone.0348289.s005.tif]
