# Supplementary material for: Rigid versus flexible: The effect of destination revitalization policy on tourists’ travel behavioral intention
Source: PLoS One. 2026 May 7;21(5):e0348289. doi: 10.1371/journal.pone.0348289 (PMC13152149; doi:10.1371/journal.pone.0348289)
Supplement: S1 File — This appendix contains summary information of Studies 1, 2, 3, 4 and 5 (Appendix A), interview outline for pilot study (Appendix B), experimental stimuli of destination revitalization policy (Appendix C), manipulation check questions and measurement items (Appendix D), summary tables of sample statistics across of Studies 1, 2, 3, 4 and 5 (Appendix E), and the data analysis of Study 1a, 1b, and 5 (Appendix F). (DOCX) [file pone.0348289.s006.docx]

**Appendix**

This appendix contains summary information of Studies 1, 2, 3, 4 and 5 (Appendix A), interview outline for pilot study (Appendix B), experimental stimuli of destination revitalization policy (Appendix C), manipulation check questions and measurement items (Appendix D), summary tables of sample statistics across of Studies 1, 2, 3, 4 and 5 (Appendix E), and the data analysis of Study 1a, 1b, and 5 (Appendix F).

**Appendix A**

Appendix A provides a comprehensive overview of all studies included in this research (see S1 Table). It systematically summarizes key methodological details across the pilot study and five main experiments, including the experimental design, hypothesis being tested, independent variable (IV) manipulation methods, dependent variable (DV) operationalization, measured or manipulated mediators/moderators, sample characteristics, and control variables for each study.

**S1 Table. Summary information of all Studies.**

| **Study** | **Experiment** | **Test** | **IV**  **manipulation** | **DV**  **operationalization** | **Mediators or moderators** | **sample** | **Control variables** |
| --- | --- | --- | --- | --- | --- | --- | --- |
| Pilot Study | Semi-structured interview | H1-H2 | / | / | / | / | / |
| Study  1a | Online  experiment | H1 | Verbal manipulation with graphics | Travel behavioral intention | None | Credamo | / |
| Study  1b | Filed  experiment | H1 | Verbal manipulation with video | Receive accommodation coupons | None | Offline reality tourists | / |
| Study  2 | Online  experiment | H1-H2 | Verbal manipulation with graphics | Travel behavioral intention | social judgment (measured) | Credamo | Age, Tourist knowledge |
| Study  3 | Filed  experiment | H1-H2 | Verbal manipulation with video | Receive accommodation coupons | social judgment (measured) | Offline reality tourists | Sense of presence, Arousal |
| Study  4 | Filed  experiment | H3-H4 | Verbal manipulation with video | Receive accommodation coupons | social judgment(measured) and temporal distance (manipulation) | Offline reality tourists | Monthly income, Level of education |
| Study  5 | Online  experiment | H3-H4 | Verbal manipulation with graphics | Travel behavioral intention | social judgment(measured) and temporal distance (manipulation) | Credamo | Travel experience, Perceived risk |

**Appendix B (Interview outline for pilot study)**

Study1- Main and mediating effects-(Qualitative Research)-Semi-structured Interviews. According to the interview theme: (1) before travel, when travelers choose to carry out the destination, after paying attention to the tourism revitalization policy of the tourist place. (2) the image judgment of the tourist place. (3) the influence on the willingness to travel and behavior Setting up the interview outline.

Interview questions:

1. On average, how many trips do you estimate you take each year?

2. Before you travel, do you learn about the information related to the tourist destination, especially the policies announced by the local government? Can you tell me what they are? (e.g., initiatives to regulate the tourism market; initiatives such as setting up special tourist buses; initiatives such as calling on citizens to give up their parking spaces for tourists, etc.)

3. Do these policies make you feel the warmth of the tourist place, or do you feel that the tourist government is more capable of governing? (Warmth vs. Competence)

4. Does knowing these policies increase or decrease your willingness to choose this tourist destination? (Travel behavioral intention)

**Appendix C (Experimental stimuli of all Studies)**

Appendix C includes all experimental materials. Stimuli for Studies 1a, 2, and 5 are shown in S1 Fig and S2 Fig. Video stimuli for Studies 1b, 3, and 4 are available at the following Bilibili links (<https://www.bilibili.com/video/BV1kj411v72Y/>; <https://www.bilibili.com/video/BV1Zw411675G/>).

**S1 Fig. Rigid policy condition stimuli of Studies 1a, 2, 5.**

**S2 Fig. Flexible policy condition stimuli of Studies 1a, 2, 5.**

**Appendix D (Measurement items of all variables)**

**S2 Table. Measurement Items.**

| **Construct** | **Measurement items** |
| --- | --- |
| **Social Judgment** | Please rate the warmth dimension that best represents your agreement of the following statements regarding the scenario you read. (1 = strongly disagree, 7 = strongly agree) |
|  | - I feel that City A is warm. |
|  | - I feel that City A is friendly. |
|  | - I feel that City A is kind. |
|  | Please rate the competence dimension that best represents your agreement of the following statements regarding the scenario you read. (1 = strongly disagree, 7 = strongly agree) |
|  | - I feel that City A is competent. |
|  | - I feel that City A is capable. |
|  | - I feel that City A is efficient. |
|  |  |
| **Traver behavioral intension** | Please rate the travel intention that best represents your agreement of the following statements regarding the scenario you read. (1 = strongly disagree, 7 = strongly agree) |
|  | - I expect to travel to the destination in the future. |
|  | - I prefer to travel to the destination than other destinations. |
|  | - If everything is as I expected, I will plan to travel to the destination. |
|  | - In the next 12 months, I may travel to the destination. |
|  | - I really want to travel to the destination. |

**S3 Table. Control Variables Measurement Items.**

| **Construct** | **Measurement items** |
| --- | --- |
| **Tourist knowledge** | Please rate your tourist knowledge that best represents your agreement of the following statements regarding the scenario you read. (1 = strongly disagree, 7 = strongly agree) |
|  | - Compared to average person, I am familiar with a wide variety of vacation destinations. |
|  | - Compared to my friends, I am familiar with a wide variety of vacation destinations. |
|  | - Compared to people who travel a lot, I am familiar with a wide variety of vacation destinations. |
| **Sense of presence** | Please rate the sense of presence that best represents your agreement of the following statements regarding the scenario you read. (1 = strongly disagree, 7 = strongly agree) |
|  | - I get the feeling that I'm there. |
|  | - It feels like the destination is right in front of me. |
|  | - The feeling of browsing this travel information is somewhat similar to your own feeling in traveling. |
|  | - The feeling of browsing this travel information is somewhat similar to your own feeling in traveling |
| **Arousal** | Please rate the arousal that best represents your agreement of the following statements regarding the scenario you read. (1 = strongly disagree, 7 = strongly agree) |
|  | - Drowsiness - Awakening |
|  | - Calm - Excited |
|  | - Failure to arouse attention - Arousing attention |
|  | - No stimulation - Stimulation |
| **Previous travel experience** | Please rate your previous travel experience that best represents your agreement of the following statements regarding the scenario you read. (1 = strongly disagree, 7 = strongly agree) |
|  | - The experience was special to me. |
|  | - The experience was favorable to me. |
|  | - The experience was valuable to me. |
|  | - The experience was meaningful to me. |
|  | - The experience was important to me. |
|  | - The experience was unique to me. |
| **Tourist risk perception** | Please rate your previous travel experience that best represents your agreement of the following statements regarding the scenario you read. (1 = strongly disagree, 7 = strongly agree) |
|  | - risk of being tricked as a tourist while travelling. |
|  | - risk of suffering any disease or infection while travelling. |
|  | - risk of inconvenient treatment while travelling |
|  | - risk of any kind of accident while travelling |
|  | - risk of suffering from a delinquency act while travelling |

**Appendix E**

**S4 Table. Summary of sample statistics for all studies.**

| **Studies** | **Study 1a** | | **Study 1b** | | **Study 2** | | **Study 3** | | **Study 4** | | **Study 5** | |
| --- | --- | --- | --- | --- | --- | --- | --- | --- | --- | --- | --- | --- |
| **Sample size** | 100 | | 87 | | 270 | | 265 | | 420 | | 500 | |
|  | n | % | n | % | n | % | n | % | n | % | n | % |
| **Gender** |  |  |  |  |  |  |  |  |  |  |  |  |
| Male | 57 | 57.0 | 54 | 62.1 | 181 | 67 | 123 | 46.4 | 140 | 33.3 | 169 | 33.8 |
| Female | 43 | 43.0 | 33 | 37.9 | 89 | 33 | 142 | 53.6 | 280 | 66.7 | 331 | 66.2 |
| **Age in years** |  |  |  |  |  |  |  |  |  |  |  |  |
| 18 to 25 | 31 | 31.0 | 55 | 63.2 | 86 | 31.9 | 54 | 20.4 | 39 | 9.3 | 53 | 10.6 |
| 26 to 35 | 52 | 52.0 | 21 | 24.1 | 120 | 44.4 | 90 | 34.0 | 146 | 34.8 | 168 | 33.6 |
| 36 to 45 | 8 | 8.0 | 10 | 11.5 | 33 | 12.2 | 108 | 40.8 | 216 | 51.4 | 246 | 49.2 |
| 46 and older | 9 | 9.0 | 1 | 1.1 | 31 | 11.5 | 13 | 4.9 | 19 | 4.5 | 33 | 6.6 |
| **Level of education** |  |  |  |  |  |  |  |  |  |  |  |  |
| Less than high school | 2 | 2.0 | 3 | 3.4 | 5 | 1.9 | 6 | 2.3 | 5 | 1.2 | 1 | 0.2 |
| High school/Technical school | 7 | 7.0 | 5 | 5.7 | 16 | 5.9 | 38 | 14.3 | 94 | 22.4 | 83 | 16.6 |
| Undergraduate/Associate degree | 62 | 62.0 | 62 | 71.3 | 188 | 69.6 | 186 | 70.2 | 275 | 65.5 | 369 | 73.8 |
| Postgraduate degree | 29 | 29.0 | 17 | 19.5 | 61 | 22.6 | 35 | 13.2 | 46 | 11.0 | 47 | 9.4 |
| **Monthly income** |  |  |  |  |  |  |  |  |  |  |  |  |
| Less than ￥3000 | 19 | 19.0 | 33 | 37.9 | 45 | 16.7 | 31 | 11.7 | 31 | 7.4 | 47 | 9.4 |
| ￥3000 to 4999 | 4 | 4.0 | 16 | 18.4 | 32 | 11.9 | 30 | 11.3 | 29 | 6.9 | 30 | 6.0 |
| ￥5000 to 6999 | 15 | 15.0 | 16 | 18.4 | 43 | 15.9 | 115 | 43.4 | 228 | 54.3 | 270 | 54.0 |
| ￥7000 to 10,000 | 27 | 27.0 | 12 | 13.8 | 56 | 20.7 | 69 | 26.0 | 113 | 26.9 | 119 | 23.8 |
| ￥10,000 or more | 35 | 35.0 | 10 | 11.5 | 94 | 34.8 | 20 | 7.5 | 19 | 4.5 | 34 | 6.8 |

**Appendix F**

### **Study 1a**

Study 1a adopted a one-factor between-subjects (rigid vs. flexible) design. A hundred respondents participated in the main experiment through Credamo.com in November 2023. Among the 100 participants, 57% were females and 43% were males, 52% were aged 26-35.

Participants will read a piece of contextual material at the beginning of the questionnaire that states, “Xiaoli is going to travel recently, she knew about the destination revitalization policy of City A through the Internet, as follows”. The two types of destination policies shown below are similar in layout and implementation elements but differ significantly in policy content (see Appendix C). For example, in terms of regulating the order of tourism security, the rigid policy reads: “Enhance the law enforcement teams, improve the quality of tourism practitioners, and safeguard tourists’ rights effectively.” The flexible policy is to “Expand the voluntary service team, improve the service level of tourism practitioners, and protect tourists’ experience effectively”.

A one-way ANOVA test was conducted to verify the main effects. The results (see S3 Fig) indicate a significant difference in participants’ travel behavioral intention (F (1, 98) = 5.76, p =0.018 < 0.05, partial η^2^ = 0.06) between rigid (M = 5.24, SD = 1.43) and flexible (M = 5.78, SD = 0.69; see Fig. 1). Hence, H1 was verified.

**S3 Fig. The influence of group type of revitalization policy on tourists’ travel behavioral intention (Study 1a).**

### **Study 1b**

Study 2b ought to verify the findings of Study 2a using a sample of real tourists. The experimental stimulus material was presented as a video (see Appendix C). The experimental procedure was similar to Study 1a, we used a one-factor (rigid vs. flexible revitalization policy) between-subjects experimental design to retest the hypotheses H1.

A total of 87 real tourists were recruited from the Chinese social media platform WeChat. Participants were asked to complete a questionnaire through an online survey platform, which randomly assigned the participants to one of the two experimental conditions (N _rigid_ = 41, N _flexible_ = 46). Among the 87 participants, 62.1% were females and 37.9% were males, 63.2% were aged 18-25 (details see Appendix E).

The act of receiving a coupon was coded as a dummy variable (1 = receive coupons, 0 = do not receive coupons). We then ran a binary logistic regression to test the main effect of destination revitalization policy on travel behavioral intention. The result revealed a significant difference (B = 1.35, Wald = 4.57, p = 0.03 < 0.05). As predicted by H1, participants in flexible revitalization policy condition (91.30%) were more likely to receiving the coupon than those who in rigid revitalization policy condition (73.17%).

### **Study 5**

Study 5 aims to evaluate the moderation of temporal distance on participants’ perceived warmth / competence and travel behavioral intention (testing H4 and H5). A 2 (rigid vs. flexible revitalization policy) × 2 (short vs. long) temporal distance between-subjects experimental design was employed.

The stimulation material of Study 5 added the description indicating temporal distance on the basis of the materials of Study 4. We also used the third person (through Xiaoli, a fictitious character) in stimulation materials to avoid the impact for social desirability. Appendix C provides more details.

A total of 500 participants (N _rigid and short_ = 121 vs. N _rigid and long_ = 121 vs. N _flexible and short_ = 134 vs. N _flexible and long_ = 124; 33.8% females, 66.2% males, 49.2% aged 36–45 years; for details see Appendix E) were recruited through Credamo.com in January 2024. The participants were randomly assigned to one of the four experimental conditions.

We conducted a 2 × 2 ANCOVA to verify the moderating effect of temporal distance. Group type of destination revitalization policy (coded as rigid = 1, flexible = 0) and temporal distance (coded as long = 1, short = 0) served as independent variables. Participants perceived warmth, perceived competence, and travel behavioral intention served as dependent variables. Previous travel experience and tourist risk perception were included as covariates. The results indicated significant interaction effect on perceived warmth (F (1, 496) = 107.91, p < 0.001, partial η^2^ = 0.18), perceived competence (F (1, 496) = 97.33, p < 0.001, partial η^2^ = 0.16) and travel behavioral intention (F (1, 496) = 277.78, p < 0.001, partial η^2^ = 0.36). Additionally, we conducted a one-way ANOVA to confirm the direction of the moderating effect of temporal distance. Under the short travel temporal distance conditions, flexible revitalization policy will generate stronger perceived warmth (M _rigid_ = 3.69, SD = 1.83; M _flexible_ = 6.23, SD = 0.51) and weaker perceived competence (M _rigid_ = 6.23, SD = 0.67; M _flexible_ = 5.74, SD = 0.78) compared to rigid revitalization policy. The travel behavioral intention of flexible revitalization policy is higher than rigid revitalization policy (M _rigid_ = 5.79, SD = 0.71; M _flexible_ = 6.20, SD = 0.45). Under the long travel temporal distance conditions, rigid policy generate stronger perceived competence (M _rigid_ = 5.41, SD = 0.92; M _flexible_ = 3.17, SD = 1.42), and weaker perceived warmth (M _rigid_ = 3.16, SD = 1.23; M _flexible_ = 3.23, SD = 1.46). The travel behavioral intention of rigid revitalization policy is higher than flexible revitalization policy (M _rigid_ = 5.33, SD = 0.86; M _flexible_ = 3.38, SD = 1.05). Therefore, H4 and H5 (see S4 Fig and S5 Fig) are supported.

**S4 Fig. Moderating effect of temporal distance between types of destination revitalization policy and travel behavioral intention (Study 5).**

**S5 Fig. Moderating effect of temporal distance between types of social judgements and travel behavioral intention (Study 5).**
