# Supplementary material for: Rigid versus flexible: The effect of destination revitalization policy on tourists’ travel behavioral intention
Source: PLoS One. 2026 May 7;21(5):e0348289. doi: 10.1371/journal.pone.0348289 (PMC13152149; doi:10.1371/journal.pone.0348289)
Supplement: Attachment — Submitted filename: Response to Reviewers.docx [file pone.0348289.s008.docx]

PLOS One Manuscript: PONE-D-25-40512

**Rigid versus flexible: The effect of destination revitalization policy on tourists’ travel behavioral intention**

We sincerely thank the editors of PLOS One and the two reviewers for their careful evaluation of our manuscript and for the constructive comments and suggestions provided. Their insightful feedback has greatly contributed to improving the clarity, rigor, and overall quality of the manuscript.

We have carefully considered each comment and revised the manuscript accordingly. All changes made in response to the reviewers’ suggestions are clearly indicated in red font in the revised version of the manuscript. Below, we provide a point-by-point response to each comment and explain how the manuscript has been revised.

**Journal Requirements**

| REVIEWER COMMENT | AUTHOR(S) RESPONSE |
| --- | --- |
| 1. Please ensure that your manuscript meets PLOS One’s style requirements, including those for file naming. | Thank you for this reminder. We have carefully reviewed the entire manuscript in accordance with *PLOS One*’s style guidelines, including requirements for formatting, structure, references, figures and tables, as well as file naming conventions. In addition, we cross-checked the manuscript against the journal’s official templates and several recently published *PLOS One* articles to ensure consistency with current editorial standards.  We hope that the revised manuscript meets the journal’s style requirements. Nevertheless, if any formatting or style issues remain, we would sincerely appreciate further guidance from the editorial office. |
| 2. We note that the grant information you provided in the ‘Funding Information’ and ‘Financial Disclosure’ sections do not match.  When you resubmit, please ensure that you provide the correct grant numbers for the awards you received for your study in the ‘Funding Information’ section. | Thank you for pointing this out. We have carefully reviewed the funding-related information and corrected the inconsistencies between the “Funding Information” and “Financial Disclosure” sections. The correct grant numbers have now been updated accordingly in the submission system, in line with your instructions. |
| 3. Thank you for stating the following financial disclosure:  “This study was supported by the National Natural Science Foundation of China (No. 72362018; 42261042), General Project of Hunan Education Department, China (No. 21C0387), and Key Project of Research project on teaching reform in ordinary higher edu-cation institutions in Hunan Province, China (No. HNJG-20230688 ).”  Please state what role the funders took in the study.  If the funders had no role, please state: "The funders had no role in study design, data collection and analysis, decision to publish, or preparation of the manuscript."  If this statement is not correct you must amend it as needed.  Please include this amended Role of Funder statement in your cover letter; we will change the online submission form on your behalf. | Thank you for this request. We have clarified the roles of the funding sources at the level of financial and institutional support, as outlined below.  The National Natural Science Foundation of China (No. 72362018; 42261042) provided funding support for the overall research project, including resources necessary for study implementation and data analysis.  The Key Project of Research on Teaching Reform in Ordinary Higher Education Institutions in Hunan Province, China (No. HNJG-20230688) supported academic exchange and dissemination related to this research.  **We have included this amended Role of the Funder statement in the cover letter as requested**. We hope that this clarification adequately addresses the journal’s requirements. |
| 4. We note that your Data Availability Statement is currently as follows: “All relevant data are within the manuscript and its Supporting Information files.”  Please confirm at this time whether or not your submission contains all raw data required to replicate the results of your study. Authors must share the “minimal data set” for their submission. PLOS defines the minimal data set to consist of the data required to replicate all study findings reported in the article, as well as related metadata and methods (https://journals.plos.org/plosone/s/data-availability#loc-minimal-data-set-definition).  For example, authors should submit the following data:  - The values behind the means, standard deviations and other measures reported; - The values used to build graphs; - The points extracted from images for analysis.  Authors do not need to submit their entire data set if only a portion of the data was used in the reported study.  If your submission does not contain these data, please either upload them as Supporting Information files or deposit them to a stable, public repository and provide us with the relevant URLs, DOIs, or accession numbers. For a list of recommended repositories, please see https://journals.plos.org/plosone/s/recommended-repositories.  If there are ethical or legal restrictions on sharing a de-identified data set, please explain them in detail (e.g., data contain potentially sensitive information, data are owned by a third-party organization, etc.) and who has imposed them (e.g., an ethics committee). Please also provide contact information for a data access committee, ethics committee, or other institutional body to which data requests may be sent. If data are owned by a third party, please indicate how others may request data access. | Thank you for this detailed guidance regarding the Data Availability requirements. We confirm that all raw data required to replicate the results reported in this study are available. (The corresponding revisions can be found in Lines 762 of the revised manuscript.)  Specifically, during the initial submission, we provided the minimal data set as a Supporting Information file in the form of an attachment entitled **“Experimental data.rar.”** This archive includes all data necessary to replicate the reported findings, including: (i) the original interview audio recordings, verbatim transcripts, and processed coding files from the Pilot Study; (ii) the raw data files from all experimental studies; (iii) the processed datasets used for analysis; and (iv) the corresponding data analysis output files.  In line with your recommendation and to further enhance data transparency and accessibility, we have also deposited the complete minimal data set in a stable public repository registered with **ICPSR**. A persistent DOI has been generated for this dataset (DOI: **https://doi.org/10.3886/E244396V1**), and this DOI has been incorporated into the revised manuscript to facilitate access for readers.  We hope that this clarification satisfactorily addresses the journal’s requirements regarding data availability. Please let us know if any additional information or adjustments are needed. |
| 5. Please include your full ethics statement in the ‘Methods’ section of your manuscript file. In your statement, please include the full name of the IRB or ethics committee who approved or waived your study, as well as whether or not you obtained informed written or verbal consent. If consent was waived for your study, please include this information in your statement as well. | Thank you for your helpful suggestion. In response, we have added a comprehensive **ethics statement** to the **Methods** section of the manuscript. This statement now includes the name of the **Ethics Committee** that reviewed and approved each study, as well as information on the informed consent process. (The corresponding revisions can be found in Lines 388, 492, 579 of the revised manuscript.)  For **Studies 2–4**, we have explicitly stated that the **study protocols, including participant recruitment, consent procedures, and experimental materials, were reviewed and approved by the Ethics Committee of the School of Tourism and Urban–Rural Planning, Jishou University**. We have clarified that **informed consent was obtained from all participants** prior to participation, and that all participation was entirely voluntary. Detailed information regarding the study's purpose, procedure, data usage, and participants' right to withdraw at any time was provided to all participants, and informed consent was given electronically before they proceeded with the study.  These revisions ensure that all ethical considerations are transparently reported in line with the journal's requirements. The updated **Methods** section now includes a full description of the ethics approval process and consent procedures for each study. |
| 6. If the reviewer comments include a recommendation to cite specific previously published works, please review and evaluate these publications to determine whether they are relevant and should be cited. There is no requirement to cite these works unless the editor has indicated otherwise. | Thank you very much for the editorial reminder. We appreciate the journal’s careful oversight and guidance throughout the review process.  We would also like to express our sincere appreciation to both reviewers for their thorough, constructive, and highly responsible evaluations of the manuscript. Their comments focused primarily on strengthening the theoretical framing, methodological rigor, and clarity of reporting, and were extremely helpful in improving the overall quality of the paper.  After carefully reviewing all reviewer comments, we confirm that neither reviewer explicitly requested the citation of specific previously published works. Therefore, the situation described in this editorial note did not arise in the present review process. Nevertheless, we have carefully updated the reference list where appropriate by incorporating recent and relevant studies to further strengthen the manuscript. |

PLOS One Manuscript: PONE-D-25-40512

**Rigid versus flexible: The effect of destination revitalization policy on tourists’ travel behavioral intention**

**Reviewer 1**

We sincerely thank you for your careful reading of the manuscript and for your constructive and thoughtful suggestions. Your comments have helped us to further refine the experimental design and strengthen the presentation of the results. We have responded to each point in detail below, with all related revisions highlighted in the revised manuscript.

| REVIEWER COMMENT | AUTHOR(S) RESPONSE |
| --- | --- |
| 1. The current form of the abstract is not well written. Author should highlight the novelty and main aim of the study. Make sure it commences with a succinct statement of the primary aim of the study, highlighting the novelty it brings to the field. This should be followed by a concise description of the research methodology employed, including the analysis techniques utilized and details regarding the sample collection process. Finally, it should conclude with a brief summary of the key findings and the implications derived from them. It is essential to emphasize the unique contributions of this study. Only highlight the most significant findings and implications to maintain focus. | Thank you for your valuable comments on the abstract. Following your suggestions, we revised the abstract to more clearly articulate the study’s primary aim, novelty, methodological approach, key findings, and main contributions, while maintaining a concise and focused structure. (The corresponding revisions can be found in Lines 20–35 of the revised manuscript.)  **Overall revision approach.**  In line with your recommendation, the revised abstract is organized to sequentially present (1) the core research aim and novelty, (2) the theoretical framing and research focus, (3) the methodological design, (4) the most salient findings, and (5) the main theoretical and practical implications. This structure is intended to improve clarity and ensure that the abstract communicates the study’s unique contribution efficiently.  **Specific revisions.**  First, to better highlight the main aim and novelty, the abstract now explicitly states that the study reconceptualizes destination revitalization policies as social signals rather than solely functional or institutional interventions. This clarification positions the study’s contribution at the outset and differentiates it from prior research that has primarily emphasized economic or governance outcomes.  Second, in response to your request for a clearer description of the research methodology, we added a concise summary indicating that the findings are based on seven studies, including one semi-structured interview study and six scenario-based experiments. This provides essential information about the empirical basis and sample collection without overloading the abstract with technical detail.  Third, to maintain focus on the most significant findings, the revised abstract emphasizes the differentiated effects of flexible versus rigid revitalization policies, the mediating roles of perceived warmth and competence, and the moderating role of temporal distance. Less central details were removed to avoid diluting the core message.  Finally, the concluding part of the abstract was revised to more clearly articulate the study’s key contributions and implications. Specifically, it highlights how the study advances destination marketing and tourism governance research by introducing a communicative perspective on policy design and by offering insights into aligning policy styles with tourists’ decision-making contexts.  We hope these revisions could directly address your concerns and improve the abstract’s clarity, coherence, and contribution to the literature. |
| 2. The introduction lacks a clear structure and focus. Please follow this suggested structure: (1) Summarize the existing issue together with its importance. (2) Explain how researchers should address the discovered areas that need further investigation. (3) The research gap must clearly relate to the existing issue. (4) The research benefits from critiquing these aspects that previous academic studies avoid. (5) Review relevant past studies. (6) Draw a contrast between this study and all related past research. (7) Clearly state the research objectives. (8) The paper details how it introduces beneficial concepts to academic literature. A properly organized introduction creates better clarity by establishing solid groundwork for the overall paper. | Thank you for your detailed guidance on improving the structure and focus of the introduction. Following your suggested framework, we reorganized the introduction into a clearly staged narrative in which each paragraph addresses a specific component of the research problem and cumulatively builds toward the study’s objectives and contributions. (The corresponding revisions can be found in Lines 37–94 of the revised manuscript.)  In the revised version, the introduction is structured as follows.  **The first paragraph** introduces the existing issue and its importance by situating destination revitalization policies within the broader context of public policy and tourism governance. It clarifies that such policies are commonly deployed in response to crises, reputational challenges, and market competition, and are traditionally discussed in terms of their functional roles in restoring tourism performance.  **The second paragraph** responds to your suggestion to explain why further investigation is needed. It emphasizes that revitalization policies are not only administrative tools but are also publicly announced and actively promoted to tourists. For example, we note that “*the Thai government introduced the ‘Trusted Thailand’ quality label in 2025 in response to safety concerns and declining visitor confidence.*” This paragraph establishes that revitalization policies operate as visible messages directed at external audiences, thereby motivating the need to understand tourists’ interpretations of these policy communications.  **The third and fourth paragraphs** collectively address the need to clearly define the research gap and critique prior research. We contrast two dominant but weakly connected research streams. On the one hand, tourism policy and destination governance studies focus on economic or institutional outcomes, such as market recovery and governance efficiency, but pay limited attention to tourists’ interpretive processes. On the other hand, research on tourist perception and destination image highlights how information cues shape evaluations and behavior, yet rarely treats public policy as a focal stimulus. As stated in the revised text, “*Taken together, these streams remain weakly connected, offering limited explanation of how tourists decode revitalization policy messages and why policy initiatives with similar objectives may generate divergent behavioral responses.*” This comparison allows the research gap to emerge directly from existing literature.  **The fifth paragraph** responds to your recommendation to draw a clear contrast between this study and prior research and to clearly state the research objectives. Here, we introduce the study’s core conceptual framing by distinguishing destination revitalization policies as rigid versus flexible and by theorizing policy designs as socially meaningful cues rather than purely institutional arrangements. This framing is grounded in the stereotype content model, which explains how social evaluations are structured along perceived warmth and competence, thereby clarifying how the study moves beyond existing economic or governance-oriented perspectives.  **The sixth paragraph** further strengthens the theoretical foundation by incorporating temporal construal theory to explain when different policy cues are likely to matter. By explaining that “*temporally distant decisions emphasize abstract, high-level considerations, whereas near-term decisions rely more heavily on concrete and feasibility-related information,*” this paragraph establishes temporal distance as a theoretically grounded boundary condition rather than an ad hoc moderator.  Finally, **the concluding paragraph** explicitly summarizes the conceptual, theoretical, and practical contributions of the study. It clarifies how the paper introduces a communicative perspective on destination revitalization policy, integrates social judgment and temporal construal theories, and offers implications for designing policy styles that align with tourists’ decision contexts.  We hope that this revised introduction closely follows the structure you recommended, strengthens the logical flow between paragraphs, and provides a clearer foundation for the study’s objectives and contributions. |
| 3. The issue is not explicitly explained and support with recent citations. | We thank you for this valuable comment. In response, we revised the Introduction to explicitly articulate the core research issue and to strengthen its support with recent and relevant citations. (The corresponding revisions can be found in Lines 44–83 of the revised manuscript.)  First, the revised Introduction now clearly defines the research issue as a disconnect between two established streams of literature. On the one hand, tourism policy and destination governance studies predominantly examine revitalization policies from economic or institutional perspectives, focusing on outcomes such as market recovery and governance efficiency (Alavi & Yasin, 2000; Solarin & Bello, 2018), while paying limited attention to how such policies are interpreted by tourists as external audiences. On the other hand, research on tourist perception and destination image emphasizes how information cues shape evaluations and behavioral intentions, yet rarely treats public policy as a focal stimulus. By juxtaposing these two streams, the revised text explicitly identifies the unresolved issue: “Taken together, these streams remain weakly connected, offering limited explanation of how tourists decode revitalization policy messages and why policy initiatives with similar objectives may generate divergent behavioral responses.”  Second, this argument is now systematically supported with integrated and up-to-date citations. The traditional functional and institutional view of tourism policies is grounded in established and recent tourism policy literature (Jamal & Camargo, 2018; Zhang et al., 2024; Higham et al., 2024). The communicative and signaling role of revitalization policies is supported by recent studies on policy communication and destination signaling (Estol & Font, 2016; Deng et al., 2019; Li et al., 2020), and is illustrated with a contemporary policy example (Thailand’s “Trusted Thailand” quality label, 2025). In addition, the revised Introduction anchors the proposed theoretical integration in well-established frameworks, including the Stereotype Content Model (Fiske et al., 2002), Temporal Construal Theory (Trope & Liberman, 2010), and governance style distinctions in public policy (Hood, 1983; Ansell & Gash, 2008).  We hope these revisions can clarify the research issue. |
| 4. The authors fail to define their research gap with clarity and supporting evidence. The authors need to present a precise identification of the unaddressed areas in past research. All necessary arguments should be presented to validate the immediate need for addressing this gap. Provide a clear reasoning that demonstrates the study’s connection to contemporary conditions. | Thank you for this comment. As this issue overlaps with the concerns raised in previous comments, We will make a brief summary of the research gap in the following contents.  In the revised Introduction, we now clearly define the research gap as the lack of understanding of how tourists interpret destination revitalization policies as communicative signals, despite the growing practice of publicly promoting such policies to tourists. We demonstrate that prior research has either focused on the economic or institutional effectiveness of revitalization policies or examined tourist perceptions without treating public policy as a focal stimulus, leaving their connection insufficiently explored. To validate the necessity of addressing this gap, we anchor the discussion in contemporary policy practice by incorporating recent citations and a current policy example, and we introduce a theoretically grounded framework to explain tourists’ interpretive processes. These revisions clarify both the gap and its relevance to current tourism governance contexts. |
| 5. Should have a standalone section for underpinning theory. This is to explain how the theory supporting the proposed paths and model. To enhance the clarity and coherence of the research, I recommend the authors thoroughly explain how or any theory support the current study and each path. By elaborating on the theory's key concepts and demonstrating its relevance to the research topic, the authors can establish a stronger connection between the theoretical foundation and the study's objectives. | We appreciate your suggestion to include a standalone theoretical section and to clarify the relevance of the selected theories to the research topic. In response, we added a new section titled “Theoretical Background”, comprising two subsections—2.1 The Stereotype Content Model and 2.2 Temporal Construal Theory. Beyond presenting these theories, this section explicitly explains why these frameworks are particularly suitable for explaining how destination revitalization policies influence tourists’ travel behavioral intentions, and how each theory supports a distinct component of the proposed model. (The corresponding revisions can be found in Lines 96–152 of the revised manuscript.)  First, the Stereotype Content Model (SCM) is introduced because the core research problem of this study concerns how tourists cognitively interpret policy signals rather than how policies perform objectively. Destination revitalization policies are publicly communicated governance initiatives, especially salient in contexts of uncertainty, crisis recovery, or image repair. From a tourist’s perspective, such policies are not directly experienced but are interpreted as indicators of the destination as a collective actor. SCM is particularly relevant in this context because it is specifically designed to explain how individuals evaluate social actors based on limited, symbolic, or second-hand information, organizing judgments along the dimensions of intentions (warmth) and capability (competence).  As explicitly stated in the revised manuscript, introducing SCM “serves a necessary theoretical function: it explains how characteristics of revitalization policies are cognitively translated into warmth and competence perceptions.” This makes SCM especially appropriate for this study, as it allows us to move beyond a functional or institutional reading of policy design and instead theorize policies as social signals that shape tourists’ judgments. Accordingly, SCM provides the theoretical foundation for the mediating mechanisms in our model, explaining how different policy styles (rigid versus flexible) influence travel behavioral intention through tourists’ warmth- and competence-based evaluations.  Second, Temporal Construal Theory (TCT) is incorporated because tourists’ travel decisions are inherently future-oriented and vary systematically with planning horizon. The same policy cue may convey different meanings depending on whether a trip is imminent or temporally distant. TCT is particularly relevant to this research topic because it explains how temporal distance alters information processing, shifting attention between abstract, goal-oriented considerations and concrete, feasibility-related concerns. In the revised manuscript, we explicitly argue that “the salience and persuasive power of different policy cues are contingent on the traveler’s planning horizon.”  This theoretical lens is especially suitable for explaining why policy styles do not exert uniform effects across all decision contexts. Rigid policies emphasize stability, institutional order, and long-term assurance, which align with high-level construals typical of distant-future decisions. Flexible policies emphasize adaptability, responsiveness, and immediate benefits, which align with low-level construals salient in near-future decisions. By grounding this logic in prior tourism research on temporal distance and information processing, TCT provides the theoretical justification for temporal distance as a moderator, specifying when the mediating effects derived from SCM become more or less influential.  In summary, the inclusion of SCM and TCT is driven by their conceptual fit with the research problem. SCM addresses the interpretive mechanism through which tourists transform policy designs into social judgments, while TCT explains the contextual condition under which different policy cues gain salience. Together, these theories establish a coherent and theoretically grounded explanation of how and when destination revitalization policies shape tourists’ travel behavioral intentions, thereby strengthening the alignment between the theoretical foundation and the study’s objectives. |
| 6. Author does not explain how the theory supporting the mediation paths and moderation paths. Please include this justification in the underpinning theory section. | Thank you for this insightful comment. In response, building on the revisions made in response to Comment 5—where two dedicated sections were added to introduce the underpinning theories and their relevance to this study—**we have further revised the hypothesis development section to explicitly articulate the theoretical foundations supporting both the mediation paths and the moderation paths**, as suggested. (The corresponding revisions can be found in Lines 199–227, Lines 235-262, Lines 269-286 of the revised manuscript.)  First, regarding the mediation mechanism, **we grounded the indirect effects of revitalization policy styles on travel behavioral intention in the stereotype content model (SCM)**. Specifically, we clarified that destination governance practices function as salient social cues that activate warmth- and competence-based social judgments. As stated in the revised manuscript, “*within the stereotype content model, governance practices function as salient social cues that activate warmth- and competence-based judgments of collective actors.*” Building on this framework, we theoretically differentiated rigid and flexible revitalization policies in terms of their dominant social signals. Rigid policies, characterized by “*rule compliance, standardization, and procedural efficiency,*” were theorized to primarily enhance perceived competence, whereas flexible policies, emphasizing “*adaptability, responsiveness, and sensitivity to stakeholder needs*,” were theorized to primarily enhance perceived warmth. We further elaborated how these differentiated social judgments reduce uncertainty, foster trust, and ultimately translate into stronger travel behavioral intentions, thereby providing a clear theoretical justification for the proposed mediation paths (H2–H2b).  Second, to address the moderation effects, **we introduced temporal construal theory as the primary theoretical lens explaining why the strength of these mediation pathways varies across temporal distance**. In the revised section, we explicitly argued that “*psychological distance systematically shapes cognitive construal,*” such that distant-future decisions rely more on abstract, high-level attributes, whereas near-future decisions emphasize concrete, situational concerns. We then linked this distinction to the inherent temporal orientation of policy styles. Rigid revitalization policies were theorized to align with distant-future, high-level construal due to their long-term, institutional, and standardized nature, thereby strengthening the competence-based mediation pathway when temporal distance is long (H3b). Conversely, flexible revitalization policies were argued to align more closely with near-future, low-level construal, amplifying the warmth-based mediation pathway when temporal distance is short (H3a).  Finally, drawing on the concept of construal fit, **we further clarified how temporal distance moderates tourists’ direct preference for different policy styles** (H4–H4b). As revised, we explained that behavioral intentions are strengthened when the characteristics of a policy style match tourists’ construal level, thereby offering an additional, theoretically grounded explanation for the moderation effects.  Overall, these revisions explicitly clarify how established theories underpin both the mediation and moderation paths in our model, we hope these revisions can address your concern and strengthening the theoretical rigor and coherence of the proposed framework. |
| 7. A proper flow for literature review as follows: (1) underpinning theory, (2) hypotheses development and (3) proposed research model. | Thank you for this helpful suggestion. We have carefully reorganized the literature review section to follow the structure you recommended. In the revised manuscript, **we first introduce the underpinning theories in two dedicated subsections**. The first subsection outlines the stereotype content model and explains how destination governance practices function as social cues that activate warmth- and competence-based judgments. The second subsection introduces temporal construal theory and elaborates on how temporal distance shapes tourists’ information processing and decision frames.  Building on these theoretical foundations, **we then present a separate hypotheses development section**. This section systematically derives the mediation hypotheses by linking revitalization policy styles to perceived warmth and competence, and further develops the moderation and moderated mediation hypotheses by integrating temporal construal theory into the proposed social judgment pathways.  Finally, **we include a proposed research model** subsection that visually summarizes the hypothesized relationships among revitalization policy styles, social judgments, temporal distance, and travel behavioral intention. This revised structure follows the suggested sequence and clarifies the logical progression from theory to hypothesis formulation and model presentation. |
| 9. Why there is a need to slit the study into five studies? | Thank you for raising this important question. The rationale for adopting a multi-study design has been explicitly clarified in both the main text and the Appendix. The purpose of splitting the investigation into multiple studies is to systematically test different components of the conceptual model and to enhance the robustness and credibility of the findings through replication across designs, samples, and materials.  As explained in the manuscript (Line 281-290), this research comprises **seven studies**, which are organized into **four sequential parts** according to their distinct theoretical and empirical purposes (see Table 1 for an outline of studies). First, a **pilot study** using semi-structured interviews was conducted as an exploratory investigation to gain initial insights into how rigid versus flexible revitalization policies are perceived and to preliminarily identify the potential mediating role of social judgments. This exploratory stage helped refine the experimental manipulations and hypotheses.  Second, **Study 1a and Study 1b** were designed to test the **main effects** of revitalization policy styles on travel behavioral intention. These two studies employ different experimental materials, samples, and control variables to replicate the main effect and reduce the likelihood that the findings are driven by idiosyncratic stimuli or sample-specific characteristics. Due to space constraints, these studies are reported in the Appendix.  Third, **Study 2 and Study 3** focus on examining the **mediating mechanisms** of perceived warmth and perceived competence. Conducting two mediation studies allows the proposed social judgment pathways to be validated across different contexts and measurement specifications, thereby strengthening confidence in the mediation effects.  Finally, **Study 4 and Study 5** extend the model by testing the **moderating role of temporal distance**. Similar to the earlier stages, two complementary studies are employed to verify the moderation effects under varied experimental settings. Study 5 is also reported in the Appendix to maintain a manageable manuscript length.  This multi-study, replication-based design follows established practices in marketing, psychology, and related social science disciplines, where multiple experiments are commonly used to validate core effects, underlying mechanisms, and boundary conditions (e.g., Su et al., 2023; Labrecque et al., 2025). Overall, splitting the investigation into multiple studies enables a step-by-step examination of the proposed model—from exploration to main effects, mediation, and moderation—while improving the robustness and generalizability of the conclusions.  [1] Su, L. , Chen, H. , Huang, Y. , & Chen, X. . (2023). In-group favoritism or black sheep effect? the moderating role of norm strength on destination residents' responses towards deviant behaviors. *Tourism Management,* *98*(000), 14.  [2] Labrecque, L. I. , Sohn, S. , Seegebarth, B. , & Ashley, C. . (2025). Color me effective: the impact of color saturation on perceptions of potency and product efficacy. *Journal of Marketing,* *89*(4). |
| 10. How do you test cause and effect relationship? | Thank you for this important methodological question. The causal relationships examined in this study are tested primarily through a **controlled experimental research design**, which is a well-established and widely adopted approach for causal inference in marketing, psychology, and related social science research (Calfee , 1985; Rahman and Islam, 2022).  First, causality is addressed through **systematic manipulation and control**. Across the studies, we employ scenario-based experiments in which participants are randomly assigned to different experimental conditions that vary only in the focal independent variable—namely, destination revitalization policy style (rigid vs. flexible), and, in later studies, temporal distance. All other aspects of the experimental scenarios, instructions, and measurement procedures are held constant. By isolating and manipulating a single factor at a time while controlling for potential confounding variables, the observed differences in outcome variables can be attributed to the manipulated independent variable. This experimental logic allows us to infer cause-and-effect relationships between policy style, social judgments, and travel behavioral intention.  Second, the **statistical analyses** employed are consistent with this experimental logic and are used to formally test the hypothesized causal effects. Specifically, **independent-sample t-tests** and **one-way ANOVA tests** are used to compare mean differences in the dependent variables across experimental conditions. These techniques are appropriate for determining whether manipulation of the independent variable leads to statistically significant differences in outcomes, thereby providing evidence for causal effects under controlled conditions.  Third, to examine the proposed mediation and moderation mechanisms, we employ the **PROCESS macro**. PROCESS is designed to estimate indirect effects, interaction effects, and moderated mediation models based on ordinary least squares regression (Hayes, 2013). By testing whether changes in the independent variable lead to systematic changes in the mediator (perceived warmth or competence), which in turn affect the dependent variable, PROCESS allows us to assess the causal pathways specified in the conceptual model. The moderated mediation analyses further test whether these indirect effects vary as a function of temporal distance, consistent with the experimental manipulations.  Overall, by combining randomized experimental designs with appropriate statistical tests for mean differences, mediation, and moderation, the study follows established methodological practices for testing causal relationships. This integrated approach enables us to draw causal inferences regarding how destination revitalization policy styles influence tourists’ travel behavioral intention through specific psychological mechanisms and under different temporal decision contexts.  We hope that these explanations adequately address your concern regarding how causal relationships are tested in this study.  [1] Calfee, R. . (1985). *EXPERIMENTAL METHODS IN PSYCHOLOGY*.  [2] Rahman, S. T. , & Islam, M. R. . (2022). Experimental method. *Principles of Social Research Methodology*, 157-165.  [3] Hayes, A. F. . (2013). *Introduction to Mediation, Moderation, and Conditional Process Analysis: A Regression-Based Approach*. |
| 11. The authors should discuss the generalizability and representativeness of their sample in relation to the target population. Authors need to clearly explain how the chosen sample is intended to be representative of and reflective of the larger population. Any strategies employed to ensure a diverse and inclusive sample should also be highlighted. This will increase the credibility of the research findings and help readers understand the extent to which the results can be generalized to the broader population. | Thank you for this important comment. In response, we have expanded the research design and procedure section to explicitly discuss the representativeness and generalizability of the samples used in this study, with detailed descriptions of participant selection, data collection procedures, and sampling strategies across studies. (The corresponding revisions can be found in Lines 313-334, 376-395, 479-502, 575-589 of the revised manuscript.)  First, in the **pilot study**, the objective was exploratory rather than statistical generalization. As stated in the manuscript, semi-structured interviews were conducted with tourists who met clearly defined eligibility criteria, including having taken at least one leisure trip within the past two years and being familiar with destination-related policy information through media exposure or personal experience. Participants were recruited using a snowball sampling technique, which is commonly employed in exploratory tourism research to access individuals with relevant travel experience and to encourage rich qualitative insights. A total of 64 tourists participated, and detailed interviewee profiles are provided in Appendix E. This stage ensured that subsequent experimental designs were grounded in tourists’ real-world perceptions rather than abstract assumptions.  Second, **Studies 2–4** employed complementary sampling strategies to enhance representativeness and generalizability. Study 2 recruited a heterogeneous pool of adult participants from a professional online survey platform that supports participant screening and random assignment, allowing access to a broad range of potential tourists with varied demographic backgrounds. Studies 3 and 4 recruited actual on-site tourists using field intercept sampling in a major tourist destination, capturing respondents who were actively engaged in real travel contexts. Together, these samples reflect both potential tourists at the planning stage and tourists at the consumption stage.  Third, several strategies were adopted to enhance diversity and robustness. These include (1) using multiple recruitment channels (online panels and field sampling), (2) collecting data across different time periods and settings, (3) ensuring random assignment to experimental conditions, and (4) replicating key effects across multiple studies with different materials and outcome operationalizations. The use of varied samples and contexts reduces the likelihood that the findings are driven by a specific subgroup or data collection method.  Overall, while the samples are not intended to statistically represent a specific national population, they are theoretically appropriate and reflective of the broader population of tourists making travel decisions. The multi-study, multi-sample design supports cautious generalization of the findings and enhances the credibility of the conclusions.  We hope that this clarification adequately addresses your concerns regarding sample representativeness, diversity, and the generalizability of the results. |
| 12. It is essential to provide a clear explanation of a sampling technique used in the study, along with the rationale for its selection. Why these techniques? The authors should describe how they utilized this sampling technique to select respondents for the survey, ensuring generalizability and representativeness towards the targeted population. By doing so, readers can better understand the methodological approach and the potential limitations associated with the sample selection. | Thank you for this valuable methodological comment. In response, we have revised the **research design and procedure** section to clearly explain the **sampling techniques used at different stages of the study, the rationale for their selection, and how they were implemented** in relation to the targeted population of tourists.  For the **pilot study**, we adopted a **snowball sampling technique**, which was selected due to the exploratory nature of this stage. As stated in the manuscript, “*a qualitative approach based on semi-structured interviews was adopted to generate preliminary empirical insights and to inform the design of subsequent scenario-based experiments*.” Snowball sampling is commonly employed in exploratory tourism research to “*access individuals with relevant travel experience and to enhance participants’ willingness to share detailed perceptions*.” Accordingly, participants were selected based on clear eligibility criteria, including having taken at least one leisure trip within the past two years and being familiar with destination-related policy information. This approach ensured that the pilot study was grounded in information-rich cases relevant to the research question, rather than aiming for statistical representativeness at this stage.  For the main empirical studies (**Studies 2–4**), we employed **controlled experimental sampling approaches**, including online panel recruitment and field intercept sampling. As described in the manuscript, Study 2 recruited participants through “*Credamo.com, a widely used professional online survey platform in China that supports randomized assignment and participant screening*,” allowing access to a heterogeneous pool of potential tourists while ensuring experimental control. Studies 3 and 4 employed “*field intercept sampling*” to recruit “*tourists who were actively visiting the destination*” in a real tourism context. These techniques were selected because they are well suited for experimental research that aims to test causal relationships while maintaining relevance to the targeted population of tourists.  Across these studies, the sampling techniques were implemented with **clear screening criteria, voluntary participation, and random assignment to experimental conditions**, as explicitly noted in the manuscript (e.g., “*participants were randomly assigned to one of the experimental conditions*”). Although these approaches do not constitute probability sampling, they are appropriate for the study’s theoretical objective of examining tourists’ psychological and behavioral responses to destination revitalization policies. Moreover, by replicating the key effects across multiple samples, settings, and sampling techniques, the study mitigates the limitations associated with any single method and supports cautious generalization to the broader tourist population.  We hope that this clarification helps explain why the chosen sampling techniques were appropriate for the research objectives, how they were applied in practice, and the potential limitations associated with the sample selection. |
| 13. Any selection criteria? | Thank you for raising this point. We acknowledge that the selection criteria were not described with sufficient clarity in the previous version of the manuscript. Following your suggestion, we have revised the **research design and procedure** section to explicitly clarify how participants were selected and what criteria were applied across the studies.  For the **pilot study**, participants were selected based on clearly defined eligibility requirements to ensure relevance to the exploratory objectives. As stated in the manuscript, participants were required to “*(1) have taken at least one leisure trip within the past two years; (2) be familiar with destination-related policy information (e.g., safety regulations, tourism recovery measures, promotional policies) through media exposure or personal experience; and (3) be willing to discuss their perceptions of destination revitalization policies and related travel intentions*.” These criteria ensured that interviewees possessed sufficient travel experience and contextual knowledge to provide meaningful qualitative insights.  For the subsequent experimental studies (**Studies 2–4**), the target population was **the general tourist population**. Accordingly, apart from basic requirements necessary for participation in experimental research, **no restrictive screening based on tourists’ demographic characteristics, travel style, income level, or destination preferences was imposed**. As described in the manuscript, participants were required only to be adults (18 years or older), to participate voluntarily, and to be able to complete the experimental tasks independently. In the field experiments, participants were additionally required to be “*visiting the destination for leisure purposes*.”  This inclusive approach was intentionally adopted to avoid over-restricting the sample and to ensure that the experimental findings are reflective of a broad range of tourists. By minimizing exclusion criteria beyond basic participation requirements, the study aims to enhance the representativeness of the sample and the applicability of the results to the wider tourist population.  We hope that this clarification adequately addresses your question regarding participant selection criteria. |
| 14. Any control variable included in this study? Why not as it could be one of the confounding variables? | Thank you for this important comment. We acknowledge that the discussion of control variables and potential confounding factors was not sufficiently clear and explicit in the previous version of the manuscript. Following your suggestion, we have revised the manuscript by adding a dedicated subsection titled “***Control variables and alternative explanations***” in each empirical study to clearly explain **which control variables were considered, why they were included, and how they were tested**. (The corresponding revisions can be found in Lines 456-469, 547-562, 645-652 of the revised manuscript.)  In **Study 2**, age and tourist knowledge were included as control variables. As stated in the revised manuscript, tourist knowledge was controlled because it “*helps reduce uncertainty and influences travel decision-making*,” while age was included given that “*age-related differences may affect individuals’ attitudes and behaviors*.” After controlling for these variables, the effects of destination revitalization policy type on perceived warmth, perceived competence, and travel behavioral intention remained significant. Additional mediation analyses further showed that neither tourist knowledge nor age exhibited a significant mediating effect, suggesting that the observed effects cannot be explained by these alternative factors.  In **Study 3**, given the use of video-based stimuli, two media-related variables—**sense of presence** and **emotional arousal**—were controlled to address potential confounding effects associated with audiovisual materials. As noted in the manuscript, sense of presence has been shown to influence perceptions and behavioral responses in virtual environments, while emotional arousal plays an important role in tourism decision-making. ANCOVA results indicated that the effects of policy type on perceived warmth, perceived competence, and travel behavioral intention remained significant after controlling for these variables. Additional PROCESS analyses confirmed that neither sense of presence nor emotional arousal served as an alternative mediating mechanism.  In **Study 4**, **monthly income** and **education level** were included as control variables to account for potential demographic influences on travel-related decision-making. A 2 × 2 ANCOVA was conducted with policy type and temporal distance as independent variables and income and education as covariates. The interaction effects between policy type and temporal distance on perceived warmth, perceived competence, and travel behavioral intention remained significant, indicating that the moderation effects are robust beyond these demographic factors.  Taken together, by explicitly incorporating theoretically relevant control variables and testing alternative explanations in each study, we have addressed potential confounding influences and strengthened the internal validity of the findings. We hope that these revisions adequately address your concern regarding the inclusion and justification of control variables. |
| 15. The procedure of data collection needs to be elaborated further. The authors should explain in more detail how they collected the data, how they approached the respondents, and how they identified participants for the survey study. This explanation should be reasonable and logical, avoiding exaggerations and providing a clear account of the steps taken in the data collection process. | Thank you for this constructive comment. We acknowledge that the data collection procedures were not described with sufficient detail and clarity in the previous version of the manuscript. Following your suggestion, we have substantially revised the **Research Procedure and Samples** sections across all studies to provide a clear, step-by-step account of how the data were collected, how respondents were approached, and how participants were identified.  For the **pilot study**, data were collected through semi-structured interviews conducted between October 6 and October 11, 2023. Given the exploratory nature of this stage, participants were recruited using a snowball sampling technique, which is commonly used in exploratory tourism research to access individuals with relevant travel experience and encourage in-depth sharing. Initial participants were drawn from the researchers’ professional and social networks and subsequently invited to recommend other eligible respondents. Clear eligibility criteria were applied (e.g., recent leisure travel experience and familiarity with destination-related policies). Interviews were conducted via telephone or in person after participants were informed of the research purpose and provided verbal informed consent. All interviews were audio-recorded with permission, transcribed verbatim, and systematically coded following established qualitative procedures.  For **Study 2**, data were collected through a scenario-based online experiment conducted between December 2023 and January 2024. Participants were recruited via Credamo, a professional online survey platform in China that supports randomized assignment and informed consent procedures. Before accessing the questionnaire, participants received detailed information regarding the study purpose, procedures, anonymity, and voluntary participation, and only those who provided electronic informed consent were allowed to proceed. Participants were randomly assigned to experimental conditions and completed the study independently online. No personally identifiable information beyond basic demographics was collected.  For **Studies 3 and 4**, data were collected through field-based experiments conducted on site in the core scenic area of Zhangjiajie. A field intercept sampling approach was adopted, whereby trained research assistants approached tourists at predefined locations (e.g., entrances, rest areas, and waiting zones) at different times of the day. Participants were first informed of the academic purpose of the study, the voluntary nature of participation, and their right to withdraw at any time without penalty. After providing verbal informed consent, participants were randomly assigned to experimental conditions, exposed to the corresponding video-based stimuli, and completed the experimental tasks and questionnaires on site. Behavioral intention was measured using real decision tasks (e.g., acceptance of accommodation or admission discount coupons), enhancing ecological validity.  Across all studies, participant recruitment, consent procedures, and data collection protocols were reviewed and approved by the Ethics Committee of the School of Tourism and Urban–Rural Planning, Jishou University. By explicitly detailing the timing, recruitment methods, consent procedures, and step-by-step data collection process for each study, we believe the revised manuscript now provides a clear, transparent, and logical account of how the data were collected.  We hope that these revisions adequately address your concern and clarify the rigor and appropriateness of our data collection procedures. |
| 16. Did you translate the questionnaire to local language? If yes, please explain how it was done. | Thank you for raising this important methodological point.  Yes, all questionnaire items were translated into the local language (Chinese). To ensure linguistic equivalence and accurate comprehension, we employed a formal back-translation procedure (Klotz et al., 2022), which is widely recommended in cross-language survey research.  Specifically, the original English measurement items were first translated into Chinese by a bilingual researcher with academic training in tourism and marketing research. A second independent bilingual researcher, who had not seen the original English version, then translated the Chinese version back into English. The back-translated English version was subsequently compared with the original items to identify any discrepancies in meaning, wording, or conceptual interpretation.  Any inconsistencies identified during this comparison were discussed jointly by the two translators and a senior researcher until consensus was reached. Minor wording adjustments were made to improve clarity and cultural appropriateness while preserving the original conceptual meaning of each item. This iterative process ensured semantic equivalence, conceptual consistency, and readability of the final Chinese questionnaire.  We believe that this formal back-translation process helped ensure that participants could accurately understand all measurement items, thereby enhancing the reliability and validity of the data collected. We hope this clarification adequately addresses your concern.  [1] Klotz, A. , Swider, B. W. , & Kwon, S. H. . (2022). Back-translation practices in organizational research: avoiding loss in translation. *The Journal of applied psychology*. |
| 17. The research instrument section is not well written. Should refer to quality paper published in high impact journal on how to report the research instruments. | Thank you for this valuable suggestion. We acknowledge that the research instrument section in the earlier version of the manuscript was not reported with sufficient clarity and rigor. Following your advice, we carefully reviewed how experimental instruments and analytical results are reported in high-impact journals.  In particular, we systematically consulted representative papers published in leading journals such as *Journal of Consumer Research* and *Tourism Management* (e.g., Ding & Kyung, 2025; Su et al., 2023) to align our reporting style with established best practices in experimental and behavioral research. Based on this benchmarking, we substantially revised the reporting of the research instruments and analytical procedures across studies.  Specifically, we improved the structure and clarity of the research instrument sections by (1) providing clearer descriptions of experimental stimuli and manipulations, (2) explicitly reporting measurement scales, item sources, and reliability indices, and (3) standardizing the presentation of manipulation checks and control variables. In addition, the reporting of analytical results was refined to more clearly distinguish between between-group comparisons (e.g., independent-sample *t*-tests and ANOVA), mediation analyses, and moderation tests, with effect sizes and statistical significance reported in a manner consistent with conventions in top-tier journals.  These revisions were applied consistently across all experimental studies to enhance transparency, methodological rigor, and readability. We believe that the revised presentation of the research instruments and results more closely follows the reporting standards of high-impact journals and substantially improves the overall quality of the manuscript. We hope that these changes adequately address your concern.  [1]Yu Ding, Ellie J Kyung, Optimizing Animation Speed: Convex Effects on Perceived Waiting Time and Digital Customer Experience, *Journal of Consumer Research*, 2025;, ucaf037, [https://doi.org/10.1093/jcr/ucaf037](https://libdb.csu.edu.cn/https/vpn/225/MSYXTLUQPJUB/10.1093/jcr/ucaf037)  [2] Su, L. , Chen, H. , Huang, Y. , & Chen, X. . (2023). In-group favoritism or black sheep effect? the moderating role of norm strength on destination residents' responses towards deviant behaviors. *Tourism Management,* *98*(000), 14. |
| 18. The reporting is confusing. | Thank you for this important comment. We acknowledge that the reporting in the earlier version of the manuscript was not sufficiently clear, which may have made it difficult to follow the logic and evidentiary flow of the study. Following your suggestion, we have substantially reorganized and clarified the reporting of methods and results in the revised manuscript.  Specifically, in the revised version, each study is now reported using a consistent and transparent structure that clearly separates research design, procedure and samples, research instruments, control variables and alternative explanations, and results. This structure allows readers to clearly understand (1) what was manipulated, (2) how data were collected, and (3) how each hypothesis was tested.  We believe that the revised reporting structure substantially reduces confusion and enables readers to more easily evaluate the validity and robustness of the findings. We hope this revised presentation adequately addresses your concern. |
| 19. For common method bias, I suggest using a full collinearity test. | Thank you for this thoughtful methodological suggestion. We appreciate your recommendation to apply a full collinearity test to assess potential common method bias (CMB). After careful consideration, we respectfully take a cautious position on this suggestion and would like to clarify our rationale.  First, the current study is based on a series of **experimental designs**, in which the independent variables (i.e., destination revitalization policy type and temporal distance) were **actively manipulated** rather than measured simultaneously with the dependent variables. Experimental methods are widely recognized as an effective approach to mitigating common method bias at the design stage, as causal inference is established through manipulation and random assignment rather than correlational associations.  Second, the full collinearity test is primarily developed and applied within the context of **structural equation modeling (SEM)** and variance-based modeling frameworks, where all constructs are treated as endogenous and assessed simultaneously. Its primary purpose is to detect multicollinearity-driven inflation that may indicate CMB in correlational, cross-sectional survey data. In contrast, our study relies on between-subjects experimental comparisons, ANOVA-based analyses, and PROCESS models, which test **causal effects** through experimental manipulation rather than shared measurement sources.  Third, the analytical logic of the full collinearity test focuses on identifying excessive correlations among latent constructs, whereas the core concern in our experiments is whether manipulating one factor leads to systematic differences in outcomes across conditions. In this sense, the test is not fully aligned with the inferential logic of experimental research, where internal validity is ensured through random assignment, controlled stimuli, and procedural separation of measures rather than post hoc collinearity diagnostics.  Importantly, to further reduce the risk of common method bias, we implemented several **procedural remedies**, including the use of experimental manipulations, varied measurement formats, behavioral outcome indicators (e.g., coupon acceptance), and the inclusion of control variables and alternative explanations across studies. These design features are commonly regarded as sufficient and appropriate safeguards against CMB in experimental research.  For these reasons, while we fully acknowledge the value of the full collinearity test in correlational and SEM-based studies, we believe that it is not methodologically necessary nor standard practice to report it in the context of the present experimental research design. We hope this explanation clarifies our position and adequately addresses your concern. |
| 20. The analysis results reporting is not clear. Author should report the preliminary analysis result (common method bias test, descriptive analysis results, and respondents profile characteristics), measurement model analysis (reliability, convergent validity, discriminant validity), and structural model analysis (path result) | Thank you for this valuable comment. We acknowledge that the reporting of analysis results in the previous version was not sufficiently clear or systematically organized. In response, we have substantially revised and standardized the reporting structure by carefully following established reporting practices in high-impact journals in marketing and tourism research. These revisions aim to improve clarity, transparency, and methodological rigor.  Specifically, in the revised manuscript, we now provide a clearer and more logical presentation of the analysis results by organizing them according to the **experimental logic of the study**, rather than a structural equation modeling (SEM) framework. Preliminary analyses, including manipulation checks, scenario authenticity tests, descriptive statistics, and respondent profile characteristics, are now explicitly reported at the beginning of each empirical study. These analyses ensure the validity of the experimental manipulations and provide readers with essential contextual information about the samples.  With regard to measurement quality, because the present research adopts a **multi-study experimental design** rather than a single SEM-based survey model, traditional measurement model assessments such as convergent validity, discriminant validity, and full common method bias tests are not fully applicable. These procedures are primarily designed for correlational survey studies in which all constructs are measured simultaneously and modeled as latent variables. In contrast, our study emphasizes **causal inference through experimental manipulation and random assignment**, and the focal constructs are analyzed using ANOVA-based methods and PROCESS models across separate studies.  Accordingly, reliability is assessed and reported using internal consistency measures (e.g., Cronbach’s α) for each scale within each study, which is consistent with standard practice in experimental research. The key causal relationships are tested through independent-sample t-tests, ANOVA/ANCOVA, and moderated mediation analyses using PROCESS, with effect sizes and confidence intervals reported to facilitate interpretation. This analytical approach directly corresponds to the study’s causal hypotheses and experimental design.  We believe that this revised reporting strategy aligns more appropriately with the methodological nature of the study and enhances the transparency and interpretability of the findings. We hope this clarification adequately addresses your concern. |
| 21. The structure of the conclusion part should be as follows: (1) Discussion, (2) Theoretical Implications, (3) Practical Implications, (4) Limitations and Future Research Recommendations, and (5) Conclusion. | Thank you for this constructive suggestion regarding the structure of the conclusion section. In response, we have carefully reorganized the concluding part of the manuscript to fully align with the structure you proposed. (The corresponding revisions can be found in Lines 654–761 of the revised manuscript.)  Specifically, the revised manuscript now adopts a clearly delineated structure titled **“Conclusions and Implications”**, which is organized into five sequential components. First, an **Overall Discussion** section synthesizes the findings across the pilot study and the subsequent experimental studies, highlighting how consistent evidence is obtained across different methods, samples, and contexts. Second, the **Theoretical Implications** section systematically elaborates on the study’s three main theoretical contributions, including its advancement of destination revitalization theory, extension of the stereotype content model to policy contexts, and integration of temporal construal into policy evaluation research. Third, the **Practical Implications** section translates the empirical findings into actionable insights for destination governance, illustrating how flexible and rigid revitalization policies serve complementary strategic functions under different temporal decision frames. Fourth, a dedicated **Research Limitations and Future Research Directions** section explicitly discusses boundary conditions and outlines avenues for future inquiry. Finally, a concise **Conclusion** section summarizes the core findings and reiterates the study’s overall contribution.  This structural revision ensures a clearer logical progression from discussion to implications and conclusion, thereby improving the coherence, readability, and academic rigor of the concluding section. We appreciate your guidance, which has helped us substantially strengthen the organization and clarity of this part of the manuscript. |
| 22. Should have a overall discussion section. | Thank you for this helpful suggestion. In response, we have added a dedicated Overall Discussion section in the revised manuscript. This section integrates and synthesizes the findings across the pilot study and the subsequent experimental studies, highlighting the consistency of results across different methods, samples, and research contexts. The inclusion of this standalone section helps clarify the overarching logic of the study and provides a coherent interpretation of the empirical findings before moving to theoretical and practical implications. (The corresponding revisions can be found in Lines 654-661 of the revised manuscript.) |
| 23. For theoretical implication, it is too shallow. This section should discuss the implications of the study's findings and how they contribute to the existing theoretical knowledge. Summarize the key findings and their relevance to the existing theoretical frameworks or models. Analyze how the findings align with or challenge current theoretical perspectives and concepts related to all the key concepts of this study. Discuss any theoretical insights or advancements that the study provides and highlight how the findings contribute to a deeper understanding of the research area. | Thank you for this insightful comment. We acknowledge that the theoretical implications in the previous version were insufficiently developed. In response, we have substantially rewritten and deepened this section to provide a more comprehensive and theory-driven discussion of how the study’s findings contribute to existing theoretical knowledge. (The corresponding revisions can be found in Lines 662-703 of the revised manuscript.)  In the revised manuscript, the **Theoretical Implications** section is no longer presented as a general summary but is explicitly organized around **three core theoretical contributions**, each corresponding to a key construct and theoretical foundation of the study. As stated at the beginning of the revised section, the discussion now focuses on clarifying how destination revitalization policies function as psychological signals, how social judgment mechanisms explain their effects, and under what temporal conditions these mechanisms are most influential. This structural reorganization ensures a clearer alignment between the empirical findings and the relevant theoretical frameworks.  First, the revised discussion clarifies the theoretical contribution to **destination revitalization and tourism governance research**. Rather than treating revitalization policies solely as institutional or structural tools, this study reconceptualizes them as *psychological and symbolic governance signals* interpreted by tourists. By shifting attention from macro-level policy outcomes to micro-level interpretation processes, this contribution helps address a long-standing limitation in governance research—namely, the lack of explanation for why policies with similar objectives may produce different behavioral responses. This perspective adds explanatory depth to existing policy frameworks by incorporating tourists’ cognitive and affective evaluations into policy effectiveness theory.  Second, the revised section strengthens the contribution to the **stereotype content model (SCM)** by extending its application to policy-driven evaluations in tourism contexts. While SCM has been widely applied to interpersonal, group, and organizational targets, how warmth and competence judgments emerge when the evaluative target is an impersonal public policy has remained theoretically underexplored. By demonstrating that rigid and flexible revitalization policies systematically activate competence- and warmth-based judgments, respectively, and that these judgments function as distinct mediating mechanisms, this study provides new theoretical insight into how fundamental social cognition processes operate in institutional settings. This extension broadens the conceptual scope of SCM and deepens its relevance for tourism and public policy research.  Third, the revised theoretical implications address a gap in **policy evaluation and tourism decision-making theory** by introducing a temporal perspective. Prior studies have largely assumed that policy effects are stable across decision stages. Drawing on temporal construal theory, this study shows that the psychological effectiveness of governance styles depends on temporal distance, thereby specifying when warmth- versus competence-based judgments are more influential. This contribution adds a dynamic dimension to policy evaluation theory and helps reconcile inconsistent findings in prior tourism studies by identifying temporal distance as an important boundary condition.  Taken together, these revisions aim to move the theoretical implications beyond a descriptive summary toward a more analytical discussion of how the findings engage with, extend, and refine existing theories. We hope that this reorganization and deeper theoretical articulation more adequately respond to your concern and contribute to a clearer understanding of the study’s theoretical value. |
| 24. For practical implications, it is not well written. The authors should provide valuable insights based on current practices and policies, supported by evidence from their research. To strengthen the practical implications, it is crucial to reference specific findings, data, or examples that demonstrate the validity and reliability of the recommendations. By incorporating this approach, the authors can offer concrete and actionable suggestions that have a solid grounding in their research findings. | Thank you for this valuable comment. In response, we have substantially revised this section to explicitly connect our empirical results to a concrete and widely observed challenge in destination governance practice. (The corresponding revisions can be found in Lines 704-731 of the revised manuscript.)  The practical problem motivating this study is that destination management organizations often face **uncertainty and inconsistency in selecting appropriate revitalization policy styles**. In practice, flexible, people-oriented policies are frequently adopted to stimulate tourism recovery, while rigid, regulation-oriented policies are sometimes criticized as suppressing market vitality. However, policymakers lack clear, evidence-based guidance on **when** and **under what conditions** each policy style is more effective. This often results in short-lived tourism booms that are difficult to sustain or, conversely, long-term governance efforts that fail to generate immediate market response.  The revised **Practical Implications** section directly addresses this problem by translating the study’s key empirical findings into policy-relevant guidance. Based on the experimental evidence that flexible revitalization policies are more effective in temporally proximal decision contexts through the activation of warmth-based judgments, we explain why such policies are particularly suitable for short-term demand activation and crisis recovery. This insight helps practitioners understand not only that flexible policies can work, but *why* they work and *when* they are most effective.  At the same time, drawing on the finding that rigid revitalization policies exert stronger effects under greater temporal distance by enhancing competence-based evaluations, the revised section clarifies the strategic value of regulation-oriented governance for long-term destination development. This addresses a common practical misconception that rigid policies are inherently detrimental to tourism growth, showing instead that their effectiveness depends on tourists’ decision time horizon.  Importantly, the study provides actionable value by reframing the choice between flexible and rigid policies as a **strategic integration problem rather than a binary decision**. By identifying the psychological mechanisms and temporal conditions underlying policy effectiveness, the findings offer destination managers a clear decision framework: flexible policies can be deployed as short-term instruments to rapidly stimulate interest and visitation, while rigid policies should anchor long-term planning, institutional credibility, and sustainable development. This evidence-based framework enables policymakers to align governance strategies with tourists’ psychological decision processes, thereby improving the effectiveness and sustainability of revitalization efforts.  Overall, the revised practical implications section is designed to demonstrate how the study addresses a concrete governance dilemma faced by practitioners and how its findings can inform more context-sensitive, psychologically grounded policy design. We hope this clearer articulation of the study’s practical value responds adequately to your concern. |
| 25. A conclusion section with not more than 150 words is required. | Thank you for this suggestion. In response, we have revised the conclusion section to ensure it is concise and focused, with a length of fewer than 150 words. The revised conclusion succinctly summarizes the core findings, highlights the key theoretical insights regarding social judgment mechanisms and temporal distance, and briefly notes the practical relevance of the study, in line with your recommendation. (The corresponding revisions can be found in Lines 747-761 of the revised manuscript.) |
| 26. Proofread the manuscript, Clarity is required, and cite more recent relevant studies. Please also check citation format and referencing style. | Thank you for this helpful suggestion. In response, we have carefully proofread the entire manuscript to improve clarity, coherence, and consistency of expression. We have revised ambiguous or lengthy sentences, improved transitions across sections, and ensured that key concepts are expressed more clearly throughout the paper. In addition, we have incorporated several recent and relevant studies to better situate our work within the current literature. Finally, all in-text citations and references have been systematically checked and revised to ensure consistency with the journal’s required citation format and referencing style. |

PLOS One Manuscript: PONE-D-25-40512

**Rigid versus flexible: The effect of destination revitalization policy on tourists’ travel behavioral intention**

**Reviewer 2**

We sincerely thank you for your thorough review and insightful comments. Your suggestions have been extremely helpful in improving the clarity, methodological rigor, and theoretical contribution of this manuscript. We have carefully addressed each of your comments, and the corresponding revisions are clearly indicated in the revised manuscript.

| REVIEWER COMMENT | AUTHOR(S) RESPONSE |
| --- | --- |
| I don’t see it as mandatory because it is already a complete piece of work, but I think that the methodological section could be strengthened if some key aspects related to the internal validity of the experimental designs were described in more detail: | Thank you very much for your thoughtful and constructive comment. We sincerely appreciate your recognition that the manuscript constitutes a complete piece of work, and we are especially grateful for your valuable suggestion regarding the internal validity of the experimental designs. Although you noted that these additions are not mandatory, we take your recommendation very seriously. All suggestions provided will be carefully considered and, where appropriate, incorporated to further strengthen the methodological rigor and clarity of the study. |
| (1) Extraneous or confounding variables: It would be helpful to identify which variables could influence the results and to explain clearly how they are controlled (for example, randomization, counterbalancing, standardized instructions, or environmental control). In the last studies, this would be especially important because the designs are becoming more complex. | Thank you very much for this insightful and constructive comment. Following your suggestion, we have systematically identified potential extraneous or confounding variables across all studies and clarified how they were controlled through experimental design, procedural standardization, and statistical techniques. (The corresponding revisions can be found in Lines 300–337, Lines 364–416, Lines 465–501 of the revised manuscript.)  First, in the **pilot qualitative study**, the primary aim was exploratory theory building rather than causal inference. Nevertheless, we minimized potential confounds by (a) applying clear participant screening criteria (recent leisure travel experience and familiarity with destination-related policies), (b) using a standardized semi-structured interview guide, and (c) adopting independent blind coding by two doctoral students with high inter-coder reliability (>95%). Disagreements were resolved through group discussion involving senior scholars, which reduced individual coder bias and ensured consistency in category assignment.  Second, in **Study 2**, a scenario-based online experiment, several design features were implemented to control extraneous variation. Participants were **randomly assigned** to rigid versus flexible policy conditions via a professional survey platform. To eliminate confounding effects of destination familiarity and prior experience, we used a **fictitious destination and hypothetical traveler**. The two policy scenarios were strictly matched in structure, length, policy domains, and presentation format, differing only in policy tone and implementation logic. Standardized instructions and a formal back-translation procedure ensured comprehension equivalence. In addition, age and tourist knowledge—two variables known to influence travel decision-making—were included as control variables, and results remained robust after accounting for these factors.  Third, in **Study 3**, where ecological validity and design complexity increased through the use of field-based video stimuli and behavioral outcomes, additional controls were introduced. Participants were **randomly assigned** to experimental conditions, watched videos on identical devices with headphones, and completed the questionnaire immediately afterward to minimize environmental noise. The two videos were carefully matched in length, narrative structure, visual style, and informational density. Moreover, two media-related variables—**sense of presence and emotional arousal**—were measured and statistically controlled to rule out alternative explanations related to audiovisual stimulation rather than policy content. The main effects and mediation results remained significant after including these covariates.  Finally, in **Study 4**, which employed a 2 × 2 between-subjects field experiment with both policy type and temporal distance manipulations, we further strengthened control strategies. Participants were **randomly assigned** to one of four conditions, and identical video stimuli from Study 3 were reused to ensure consistency. Temporal distance was manipulated using well-established time frames from prior research. To address potential socioeconomic confounds in real-world decision-making, **monthly income and education level** were included as covariates. The key interaction effects on perceived warmth, perceived competence, and behavioral intention remained significant after controlling for these variables.  Overall, across studies we employed a combination of randomization, standardized stimuli and instructions, environmental matching, validated measurement scales, and covariate controls. We hope that these revisions and clarifications adequately address your concerns regarding extraneous and confounding variables and convincingly demonstrate the internal validity of our findings, especially in the later and more complex experimental designs. |
| (2) Controls used to address threats to internal validity: such as history, maturation, selection, and experimenter effects. It would be useful to indicate which procedures were used to reduce these threats. | Thank you very much for this helpful comment. In response, we have clarified how threats to internal validity were addressed primarily through **procedural controls embedded in the experimental design**, rather than relying solely on post hoc statistical adjustments. Specifically, we structured the experimental scenarios, recruitment process, manipulation delivery, and control variable selection to isolate the focal effects of destination revitalization policy and reduce interference from irrelevant factors. (The corresponding revisions can be found in Lines 444-455, Lines 529–537, Lines 627–634 of the revised manuscript.)  First, to minimize contamination from prior knowledge, historical context, and real-world destination images, we deliberately employed **fictitious experimental settings**. As stated in the revised manuscript, *“the experimental stimuli described a fictitious destination (‘City A’) and a hypothetical traveler (‘Xiaoli’) to avoid confounding effects of prior destination familiarity or real-world experiences.”* By instructing participants to base their judgments solely on the provided information, this design reduced history-related and experience-based biases.  Second, **participant recruitment and assignment procedures** were designed to reduce selection-related threats. In Study 2, participants were recruited via a professional online platform that *“supports randomized assignment and participant screening,”* and were randomly allocated to rigid versus flexible policy conditions. In the field experiments (Studies 3 and 4), tourists were recruited using intercept sampling with clear eligibility criteria, and *“participants were randomly assigned to one of the experimental conditions on site.”* These procedures helped ensure comparability between groups before manipulation.  Third, **experimental manipulations were delivered in a highly standardized manner** to reduce experimenter effects. Across studies, policy information was conveyed through written scenarios or pre-recorded videos rather than verbal explanations. In Studies 3 and 4, the manuscript explicitly notes that *“the two videos were matched in terms of length, narrative structure, presentation style, and informational density,”* and participants viewed the materials on identical devices following unified instructions. This procedural standardization limited unintended cues from experimenters and ensured consistency across conditions.  Fourth, to further isolate the focal effects, we incorporated **theoretically grounded control variables** into the experimental procedure and analyses. As described in the manuscript, age and tourist knowledge were controlled in Study 2, while sense of presence and emotional arousal were controlled in Study 3 due to the use of video stimuli. In Study 4, monthly income and education level were included to account for socioeconomic influences on travel-related decision-making. Importantly, the main and interaction effects remained significant after including these controls, indicating that the findings were not driven by alternative explanations.  Overall, through the combined use of fictitious scenarios, randomized assignment, standardized manipulation delivery, and carefully selected control variables, we sought to minimize threats to internal validity at the procedural level. We hope that the above clarifications and revisions adequately address your concerns and that these efforts can satisfactorily resolve the issues you raised. |

**General response to the reviewers’ comments:**

We greatly appreciate the reviewers’ constructive feedback and time spent reviewing our paper. The revision process has been valuable in providing food for thought and contributing to a higher-quality submission.

Many thanks again,

The authors
