# Supplementary material for: Rigid versus flexible: The effect of destination revitalization policy on tourists’ travel behavioral intention
Source: PLoS One. 2026 May 7;21(5):e0348289. doi: 10.1371/journal.pone.0348289 (PMC13152149; doi:10.1371/journal.pone.0348289)
Supplement: Attachment — Submitted filename: Response_to_Reviewers_auresp_2.docx [file pone.0348289.s009.docx]

PLOS One Manuscript: PONE-D-25-40512

**Rigid versus flexible: The effect of destination revitalization policy on tourists’ travel behavioral intention**

We sincerely thank the editors of PLOS One and the two reviewers for their careful evaluation of our manuscript and for their constructive comments throughout the review process.

We are grateful for the positive feedback and recognition received during both rounds of review. The reviewers’ and editors’ comments have helped us improve the manuscript and ensure its clarity and quality.

Below, we provide a point-by-point response to all comments and explain the corresponding revisions made in the manuscript. All changes are marked in red in the revised version.

| **Journal Requirements** | |
| --- | --- |
| REVIEWER COMMENT | AUTHOR(S) RESPONSE |
| 1. If the reviewer comments include a recommendation to cite specific previously published works, please review and evaluate these publications to determine whether they are relevant and should be cited. There is no requirement to cite these works unless the editor has indicated otherwise. | Thank you for this helpful note.  We would like to sincerely express our appreciation to both reviewers for their careful, thorough, and responsible evaluations. Their comments were detailed and constructive, and have been very helpful in improving the manuscript.  I declare that **Neither reviewer suggested citing specific previously published works**. |
| 2. Please review your reference list to ensure that it is complete and correct. If you have cited papers that have been retracted, please include the rationale for doing so in the manuscript text, or remove these references and replace them with relevant current references. Any changes to the reference list should be mentioned in the rebuttal letter that accompanies your revised manuscript. If you need to cite a retracted article, indicate the article’s retracted status in the References list and also include a citation and full reference for the retraction notice. | Thank you very much for this valuable suggestion.  Following the journal’s guidelines, **we have carefully and systematically reviewed the reference list**. During this process, we identified several minor errors and formatting inconsistencies, which have now been thoroughly corrected (e.g., Lines 809, 870, and 947; all revisions are highlighted in red in the “Revised Manuscript with Track Changes”).  In addition, we have double-checked the accuracy of all references, including citation formats and links. **We confirm that none of the cited works have been retracted**. Compared with the previous version, the revised manuscript involves only formatting corrections to the reference list, without any addition or removal of references.  We sincerely appreciate the editorial office’s guidance, which has helped us further improve the accuracy and consistency of the manuscript. We would be grateful for any further comments or suggestions you may have. |
| **Reviewer 1** | |
| REVIEWER COMMENT | AUTHOR(S) RESPONSE |
| I am satisfied with the revised manuscript. All my comments are addressed sufficiently. Thus, I have no further comments. | We sincerely thank you for the time and effort devoted to our manuscript. Your previous comments have been highly valuable and have greatly contributed to improving the quality of this work. We have also learned a great deal through the revision process.  We are truly grateful for your positive evaluation of the revised manuscript, and wish you all the best in your future research endeavors. |
| **Reviewer 2** | |
| REVIEWER COMMENT | REVIEWER COMMENT |
| Congratulations on the rigor of your research;  Please, in terms of style, correct the reference error on line 203 and label the figures as “Author's own work”. | We sincerely thank you for your positive evaluation of our work and for your careful and rigorous review. Your comments are very helpful and have improved the accuracy and presentation of the manuscript.  **Regarding the comment “correct the reference error on line 203,” we have corrected the issue**. The original problem was an incorrect URL link in the reference entry. The link has now been replaced with the correct source, and the corresponding reference information has been updated accordingly.  In addition, **we carefully checked all in-text citations and the reference list**. We found several formatting and consistency issues, such as missing page numbers, cases where multi-author references did not display the first six authors as required, and minor citation style inconsistencies. These issues have now been fully corrected. The revised parts are marked in red in the “Revised Manuscript with Track Changes” (e.g., Lines 809, 870, and 947).  Regarding your suggestion to label the figures as “Author’s own work,” **we have added this statement in the captions of all five figures**. The statement has been clearly included in each figure note to indicate authorship (see Lines 304, 363, 448, 633, and 636).  We sincerely appreciate your careful review. Your suggestions have significantly improved the clarity, accuracy, and overall quality of the manuscript. We would be grateful for any further comments or suggestions you may have. |

**General response to the reviewers’ comments:**

We greatly appreciate the reviewers’ constructive feedback and the time spent reviewing our manuscript. We also sincerely appreciate the positive evaluation and recognition of our work in this revision.

Many thanks again,

The authors
